# RNA-Seq Identification of Cd Responsive Transporters Provides Insights into the Association of Oxidation Resistance and Cd Accumulation in *Cucumis sativus* L.

**DOI:** 10.3390/antiox10121973

**Published:** 2021-12-10

**Authors:** Shengjun Feng, Yanghui Shen, Huinan Xu, Junyang Dong, Kexin Chen, Yu Xiang, Xianda Jiang, Chenjie Yao, Tao Lu, Weiwei Huan, Huasen Wang

**Affiliations:** 1 Collaborative Innovation Center for Efficient and Green Production of Agriculture in Mountainous Areas of Zhejiang Province, College of Horticulture Science, Zhejiang Agriculture and Forestry University, Hangzhou 311300, China; 20170039@zafu.edu.cn (S.F.); xuhuinan0625@stu.zafu.edu.cn (H.X.); dongjunyang@stu.zafu.edu.cn (J.D.); 2017701492004@stu.zafu.edu.cn (K.C.); 201901070134@stu.zafu.edu.cn (Y.X.); jiangxianda@stu.zafu.edu.cn (X.J.); ycjdyx@stu.zafu.edu.cn (C.Y.); 2 State Key Laboratory of Subtropical Silviculture, Laboratory of Plant Molecular and Developmental Biology, College of Forestry and Biotechnology, Zhejiang Agriculture and Forestry University, Hangzhou 311300, China; shenyanghui0610@stu.zafu.edu.cn; 3 Institute of Vegetables and Flowers, Chinese Academy of Agricultural Sciences, Beijing 100081, China; lutao@caas.cn; 4 College of Chemistry and Materials Engineering, Zhejiang Agriculture and Forestry University, Hangzhou 311300, China; 20130074@zafu.edu.cn

**Keywords:** cucumber, cadmium, ROS, transporter, Cd accumulation, Cd tolerance

## Abstract

Greenhouse vegetable production (GVP) has grown rapidly and has become a major force for cucumber production in China. In highly intensive GVP systems, excessive fertilization results in soil acidification, increasing Cd accumulation and oxidative stress damage in vegetables as well as increasing health risk of vegetable consumers. Therefore, enhancing antioxidant capacity and activating the expression level of Cd transporter genes seem to be feasible solutions to promote plant resistance to Cd stress and to reduce accumulated Cd concentration. Here, we used transcriptomics to identify five cucumber transporter genes (CsNRAMP1, CsNRAMP4, CsHMA1, CsZIP1, and CsZIP8) in response to cadmium stress, which were involved in Cd transport activity in yeast. Ionomics, gene expression, and REDOX reaction level association analyses have shown that the transcript of CsNRAMP4 was positively correlated with Cd accumulation and antioxidant capacity of cucumber roots. The expression level of CsHMA1 was negatively correlated with Cd-induced antioxidant capacity. The overexpression of CsHMA1 significantly relieved Cd stress-induced antioxidant activities. In addition, shoots with high CsHMA2 expression remarkably presented Cd bioaccumulation. Grafting experiments confirmed that CsHMA1 contributed to the high antioxidant capacity of cucumber, while CsHMA2 was responsible for the transport of Cd from the roots to the shoots. Our study elucidated a novel regulatory mechanism for Cd transport and oxidative damage removal in horticultural melons and provided a perspective to regulate Cd transport artificially by modulating Cd accumulation and resistance in plants.

## 1. Introduction

Greenhouse vegetable production (GVP), a main part of vegetable production in China, has grown rapidly. Chinese GVP areas reached 4.1 million ha in 2021 [1,2]. In highly intensive GVP systems, excessive fertilization has resulted in heavy metal accumulation and soil acidification, which, in turn, has increased heavy metal accumulation in vegetables and has posed health risks to vegetable consumers [3,4]. Anecdotal evidence suggests that heavy metals in protected cultivated soil shows a significant accumulation trend, and the content of heavy metals was significantly higher than that in open cultivation soil [5]. Protected vegetable plots have become the type of vegetable plot with more serious accumulation of heavy metals after industrial and mining/sewage irrigation plots, which should be paid with attention. The Cd content in protected farmland was found to be the most serious, and the Cd contents in the south, north, and northwest of China were 41.7%, 54.5% and 11.1%, respectively, of the total Cd contamination of land [5].

Cucumber is the dominant staple vegetable plant in many countries of the world. China produces 187 million tons of cucumber annually, accounting for approximately 35% of global production [6]. The quality and safety of cucumber are therefore important considerations in China. However, soils for cucumber culture have been contaminated by Cd, and this phenomenon has been a public concern in China. The increase in mining and industrial activities has also raised food safety issues produced by excessive Cd emission. Accordingly, reducing Cd concentration and REDOX states in cucumber is necessary to decrease the risk posed to humans by contaminated fruits [3]. Among the many remediation methods of Cd pollution, developing a biotechnology for manipulating Cd uptake and transduction in plants is beneficial to reduce environmental and health risks.

Cd transduction is controlled by a group of metal transporters [7]. Given that no specific Cd transporter is characterized in plants, Cd is absorbed into plants through a broad range of cation transport systems. To date, many Cd carriers in plants have been functionally identified. In plants, many transporters for divalent transition metals have Cd^2+^ uptake activities. For instance, AtIRT1, a ZIP family transporter for Fe^2+^, Zn^2+^, and Mn^2+^, mediates Cd uptake in the roots of Arabidopsis thaliana [8,9]. OsNramp5 (natural resistance-associated macrophage protein 5) is the major transporter of Cd uptake in rice [10]. Cd must be transported from root uptake through the xylem to shoot tissues in many plants [11]. P1B-type ATPase (OsHMA3) has been identified as a regulator for xylem Cd transport in rice by mediating vacuolar sequestration of Cd in root cells [12,13]. Furthermore, the overexpression of HMA3 enhances the tolerance and accumulation of Zn and Cd in Arabidopsis thaliana WS background [14]. CsHMA1 in the tonoplast of cucumber is responsible for Cd and Pb tolerance [15]. Loading of Cd^2+^ into the xylem and transportation from the root to shoot are mediated by ATPases HMA2 and HMA4, respectively [7,16]. Similar to HMA2, CsHMA2 could secrete Cd out of the plasma membrane in cucumber root tissues [15]. The Cd transporter OsLCT1 mediates xylem-to-phloem transfer in node 1. The downregulation of the OsLCT1 gene directly affects the reduction of Cd in grains [17]. The above-mentioned genes are responsible for long-distance transport of Cd. Many other genes involved in Cd accumulation and tolerance have also been reported in plants. For example, lower Cd accumulation in plants can be achieved by Cd secretion, which is driven by a group of Cd efflux transporters, such as OsHMA9 [18], OsCAL1 [19] OsZIP1 [20], and OsCTF [21]. Furthermore, several metallochaperones, such as OsHIPP29, OsHIPP42, and OsHMP, are actively partitioning Cd allocation and detoxification [22,23,24]. Although several Cd transporters have been identified in plants, few studies have been conducted on horticultural crops, especially in cucumbers. Therefore, the excavation of Cd absorption or transport proteins in cucumber by modern molecular biology and multiomics is important because clean cucumber production is critical for sustainable agriculture.

ROS is a toxic and signal material caused by the stress of heavy metals. Heavy metals (like Cd) can produce ROS via the Fenton and Haber–Weiss reactions and indirectly inhibit the activities of antioxidant enzymes [25]. In particular, H_2_O_2_ acts as a signaling molecule in response to heavy metals and other stresses [10,26,27,28,29,30]. H_2_O_2_ levels increase in response to Cu and Cd treatment in A. thaliana upon Hg exposure in tomato [31] and in response to Mn toxicity in barley [31]. Modification of these ROS activities may confer plant tolerance or sensitivity to metal stress [22,23]. Plants respond to excessive heavy metals in environments by adjusting their physiological and molecular machineries regulated by global gene expression. Recent global profiling of transcriptome analysis identified a large number of Cd responsive genes involved in ROS signal transduction [32,33]. Relevant signal transduction pathways include Ca–calmodulin system, hormones, and mitogen-activated protein kinase (MAPK) phosphorylation cascade, which all converge by activating Cd-related ROS genes [10,27,28,34]. However, the complex regulatory mechanisms for the processes remain largely unknown. Cd transporters play an important role in Cd accumulation and tolerance in plants, but limited information is known about whether these tissue- and cell-specific transporters are involved in the regulation of ROS signaling. Our previous studies have found that changes in some Cd transporter activities can affect botanic response to Cd stress-induced oxidative stress [22,23], suggesting that plant Cd transporters are involved in ROS signal responses. The detailed mechanism must be further clarified.

In this study, we adopted RNA sequencing (RNA-Seq) to identify five Cd responsive metal transporters in Cd-exposed cucumber seedlings. Yeast metal transport experiments have shown that the genes can transport Cd. Ionomics, gene expression profile, and oxidative stress correlation analysis showed that CsNramp4 in cucumber was correlated with Cd uptake in roots and high ROS oxidation level in seedlings. Meanwhile, CsHMA3 was correlated with low ROS oxidation level in seedlings. In addition, CsHMA2 was involved in the transport of Cd from the roots to the shoots of cucumber and had affected the accumulation of Cd in the fruits. This work highlights the importance of metal transporters in plant responses to oxidative stresses from Cd. These transporters may be used to reduce Cd accumulation and the damage of oxidative stress in cucumber, thereby preventing the environmental risks of Cd to human health through the food chain.

## 2. Materials and Methods

### 2.1. Plant Cultivation and Treatment

Cucumber inbred line ‘R1461′ was provided by Prof. Zhang Xiaolan from China Agricultural University (Beijing, China). Twenty-two cucumber cultivars were purchased from Kerun Cucumber Research Institute in Tianjin. Plump cucumber seeds were selected and placed into a sterilized Petri dish with filter paper to germination. After germinating, the seedlings were cultured in a Petri dish for 3 days. The seedlings with the same growth rates were selected, and the hypocotyls were fixed with a sponge and placed into a 1 L black beaker containing Yamazaki nutrient solution for hydroponics for 10 days. The seedlings were treated with 0, 1, 10, 50, and 150 μM CdSO_4_ for 48 h to 20 days, and the nutrient solution was renewed every 2 days. Three independent biological replicates were performed for each treatment. The treated seedlings were prepared for primary root length, fresh weight, Cd content, oxidative stress level, and gene expression.

### 2.2. RNA Extraction and Gene Expression Analysis

The treated seedlings and their control counterpart were used to isolate total RNA by TRIzol reagent (Ambion, Carlsbad, CA, USA). Approximately 1 μg of the RNA was treated with DNaseI. EasyScript One-Step gDNA removal was followed by the synthesis of cDNA by using SuperMix (TransGen, Beijing, China) based on the manufacturer’s protocol. qRT-PCR analysis was performed in 18 μL reaction solution containing cDNA (2 μL), 2 × SYBR Premix Ex Taq (Takara, Japan) (10 μL), and 200 nM primers. The reaction was carried out under denaturation at 95 °C for 30 s for one cycle, followed by 40 cycles of denaturation at 85 °C for 5 s and 50 °C for 34 s for extension. Actin1 and Ubiquitin10 were used as internal controls with specific primers.

### 2.3. Transcriptome Analysis

Ten-day-old cucumber seedlings were exposed to 0 and 50 μM Cd for 4 d and were sampled. Total RNA from Cd-treated (+Cd) and Cd-free (Normal) cucumber seedlings was isolated using the TRIzol Reagent (Ambion, Carlsbad, CA, USA) and pooled for RNA sequencing. The extracted RNA was treated with DNaseI (Qiagen, Dusseldorf, Germany) at 25 °C for 30 min. mRNA was purified with oligo (dT)-rich magnetic beads and broken into short fragments. First- and second-strand cDNAs were synthesized. cDNAs were end-repaired and phosphorylated using T4 DNA polymerase and Klenow DNA polymerase. The Illumina paired-end solexa adaptors were ligated to cDNA fragments. The ligated products were purified on a 2% agarose gel. Six libraries (+Cd1, +Cd2, +Cd3, Normal1/Contorl1, Normal2/Contorl2, Normal3/Contorl3) were sequenced using Illumina hiseq2500. The original image data generated by the sequence providers were transferred into nucleotide sequences by base calling and defined as raw reads. All subsequent analyses were performed on high-quality clean read datasets according to bioinformatics analysis approach summarized in Supplementary Table S1. Htseq-count [35] software was used to obtain the number of reads compared with genes in each sample. CuffLinks [36] software was used to calculate the FPKM (fragments per kb per million read) value of gene expression [37]. False discovery rate (FDR) was used for multiple test correction of *p* values. In this study, FDR ≤ 0.05 was used as the threshold to determine the significance of gene expression variation.

### 2.4. Bioinformatics Analysis of Cucumber Cd-Responsive Transporters

The structures of the transporter sequence on the protein sequences were downloaded from the cucumber (http://cucurbitgenomics.org/, accessed on 12 July 2021), rice (http://rice.uga.edu/, accessed on 12 July 2021), and *Arabidopsis* (https://www.arabidopsis.org/, accessed on 12 July 2021) Genome Database. Sequence comparison and evolutionary tree analysis were carried out using MEGA6.0 software (Mega Limited, Auckland, New Zealand). The neighbor-joining phylogeny of these sequences was constructed with 1000 bootstrap replicates [38].

### 2.5. Analysis of Metal Quantification

Cucumber and *Arabidopsis* seedlings with similar growth rates under Cd treatment were selected, cleaned three times with deionized water and 2.5 mM CaCl_2_ water solution, and dried at 75 °C for 2 days. The tissue samples were weighed, and then digested in 5 mL of nitric acid. The concentration of metals in the digested solution was quantified by Inductively Coupled Plasma–Atomic Emission Spectrometry (ICP-AES) (Optimal 2100 DV, PerkinElmer Instruments, Waltham, MA, USA) [39].

### 2.6. Determination of MDA, H_2_O_2_, and Lipid Peroxidation

The seedlings of 22 cucumber cultivars and transgenic *Arabidopsis* were grown hydroponically for 10 d and transferred to the same culture solution containing 0, 10, or 50 μM Cd for 48 h. H_2_O_2_ content was quantified as described previously [39]. One gram of cucumber or *Arabidopsis* seedlings was extracted in 1 mL of 80% ethanol. In brief, 100 μL of the plant extracts were incubated for 30 min with 1 mL of solution containing 90% methanol (*v*/*v*), 25 mM H_2_SO_4_ (*v*/*v*), 250 μM ferrous ammonium sulfatehexahydrate, and 100 μM xylenol orange. The absorbance of the homogenate was recorded at 560 nm. Standard curves ranging from 0 to 200 μM were established and used to calculate H_2_O_2_ concentration. MDA content was measured using the method described by Heath and Packer (1968) with slight modification. The cucumber and *Arabidopsis* seedlings (0.1 g) were ground in 1 mL of 10% (*w*/*v*) trichloroacetic acid. After centrifugation at 12,000× *g* for 10 min at 4 °C, the supernatant was collected, and 2 mL of the supernatant fraction was mixed with 2 mL of 0.6% TBA solution. The mixtures were heated at 95 °C for 30 min and then cooled quickly in an ice bath. The resulting mixtures were centrifuged at 10,000× *g* for 10 min, and the absorbance of the supernatants was recorded at 450, 532, and 600 nm.

### 2.7. Association Analysis of Ionomics and Gene Expression Profiles

To explore the association between Cd accumulation in the natural populations of cucumber and the transporters mentioned above, we selected 23 cucumber cultivars collected throughout China and cultured them in soils with simulated Cd pollution conditions (0.5 μM) in facilities. Cd content was measured in both shoots and roots.

### 2.8. Grafting Experiment

To investigate the physiological role of CsNramp4 and CsHMA2 in plants, we designed a series of grafting experiments to verify their function. We selected four widely cultivated cucumber varieties (*Jinyou12*, *Jinyou49*, *Jinmei3*, and *R1461*). Ten-day-old cucumber plants were selected for grafting. After 7 days of growth in Yamazaki nutrient solution, the live seedlings were supplemented with 0 and 0.5 μM Cd for 48 h (determination of Cd content) and 4 days (gene expression detection).

To study the direct correlation between oxidative stress level and *CsHMA1* and *CsNramp4* ex pression in cucumber, we selected varieties *Jinyou 1*, *Jinyuan 11*, *Jindong F6*, and *Jinyou 315* as rootstock and scion for grafting. Within 10 days of Cd treatment, Cd accumulation in cucumber plants was determined. Ten-day-old cucumber plants were selected for grafting. After 7 days of growth in Yamazaki nutrient solution, the live seedlings were supplemented with 0 and 0.5 μM Cd for 48 h (ROS detection) and 4 days (gene expression detection).

### 2.9. Plant Expression Vector and Agrobacterium-Mediated Transformation of Arabidopsis

pSY06 was used as an expression vector with ubiquitin 10 promoter [40]. CsNramp4 and CsHMA1 CDS sequences were homologous and recombined into the pSY06 vector. The confirmed clones were transformed into *Agrobacterium tumefaciens* strain GV3101 and then into *Arabidopsis* by floral dip method [39]. Positive transgenic lines were selected on the soil with 50 mg L^−1^ basta. Ten independent UBI10:CsNramp4 and 15 UBI10:Cs CsHMA1 transgenic lines (single copy and homozygous line) were obtained. Five lines were randomly selected for transcription analysis by qRT-PCR, and two of them were used for functional characterization.

### 2.10. Subcellular Localization of CsNramp1/4

The coding sequences of CsNramp1 and CsNramp4 were amplified by RT-PCR and inserted into pCAMBIA1300-GFP vectors driven by the 35S promoter. The CsNramp1 and CsNramp4-GFP fusion vector was transformed into *Arabidopsis* leaf mesophyll protoplasts [24]. Fluorescence was visualized using confocal laser scanning microscopy (Olympus, Japan).

### 2.11. Yeast Complementation Assay

The cDNA fragments containing an entire open reading frame of CsNramp1, CsNramp4, CsZIP1, and CsZIP8 were amplified. The fragments were cloned into a pYES2 vector. The resulting plasmids were transformed into the mutant yeast strain *ycf1* (Cd-sensitive strain [41]). *ycf1* complementation by drop-spotting assays was performed on synthetic defined (SD)-Ura medium containing 2% galactose, 0.67% yeast nitrogen base (Sigma), 2% agar [41], and supplemented with 50 or 60 μM Cd.

### 2.12. Statistical Analysis

Results were presented by means of three independent replicates (*n* = 3), and each replicate contained at least 10 plants. Data between different treatments were statistically analyzed by ANOVA, followed by means separation by least significant difference (LSD) test if the ANOVA result is significant at *p* < 0.05. Data were analyzed using SPSS 22 (IBM SPSS, Chicago, IL, USA).

## 3. Results

### 3.1. Growth and Physiological Responses of Cucumber to Cadmium Stress

**Figure 1:**
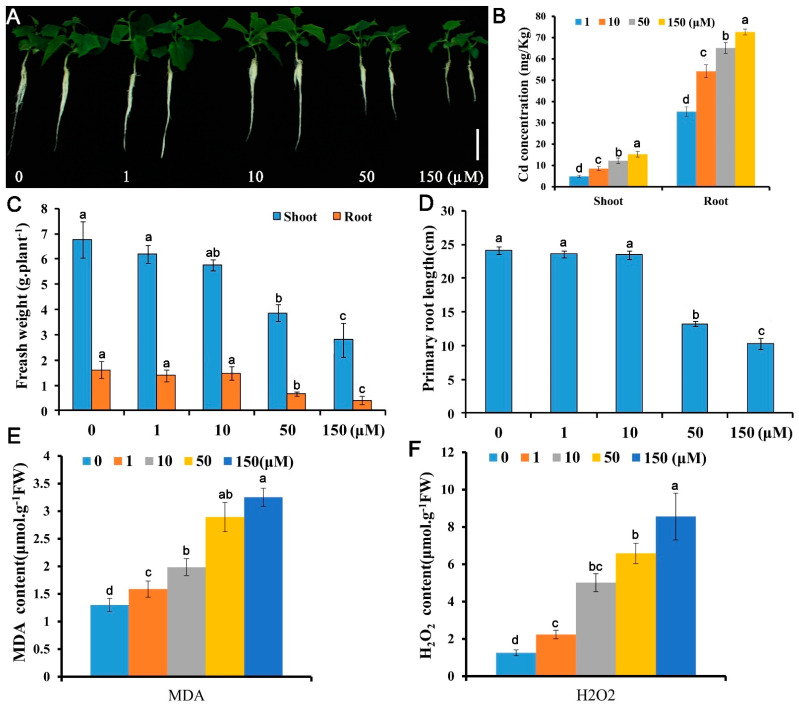
Growth and physiological responses of *Cucumis sativus* L. to Cd stress. Ten-day-old seedlings were treated with 0, 1, 10, 50, or 150 μM CdSO4 for 10 d (**A**–**D**) or 48 h (**E**,**F**) and growth and physiological indices were assessed: (**A**) morphology; (**B**) Cd content; (**C**) fresh weight; (**D**) primary root elongation; (**E**) MDA content; (**F**) H_2_O_2_ content. Vertical bars represent standard deviation of the mean of three replicates (*n* = 10–20 seedlings). Significance of differences between the treatments was statistically evaluated by analysis of variance (ANOVA). Different letters on the bars indicate significant difference (*p* < 0.05) between the treatments.

Five concentrations (0, 1, 10, 50, and 150 μM) were employed to test the growth responses of *Cucumis sativus* seedlings to Cd stress. Growth indicators in terms of fresh biomass and primary root elongation decreased with Cd concentrations application (Figure 1A–D). The fresh weights for 1, 10, 50, and 150 μM Cd exposed seedlings were 46.1%, 31.1%, and 17.0% of the control (0 μM Cd) and the primary root lengths were 73.0%, 52.4%, and 47.6% of the control, respectively. Both the Cd contents in the cucumber shoots and roots under Cd stress were significantly increased compared with the control (Figure 1B). Cd overload in plant cells is oxidative toxic [25]. To investigate the REDOX responses of cucumber root to Cd stress, malonyldialdehyde (MDA) and H_2_O_2_, two oxidative stress indicators was detected in roots [39,42]. Similar to the growth results, the oxidative stress increased gradually with Cd concentration and increased rapidly at 50 μM Cd toxicity (Figure 1E,F). These data suggest that *Cucumis sativus* plants could be more sensitive to Cd at 50 μM, leading to 40–60% reduction in biomass, 1.8–2.5-fold change increase in Cd concentration, and 3.5–5.3-fold change increase in the H_2_O_2_ content compared with the control. Therefore, this concentration was used to identify Cd response genes.

### 3.2. Identification of Cucumis sativus Coding-Transcripts in Response to Cd Stress

**Figure 2:**
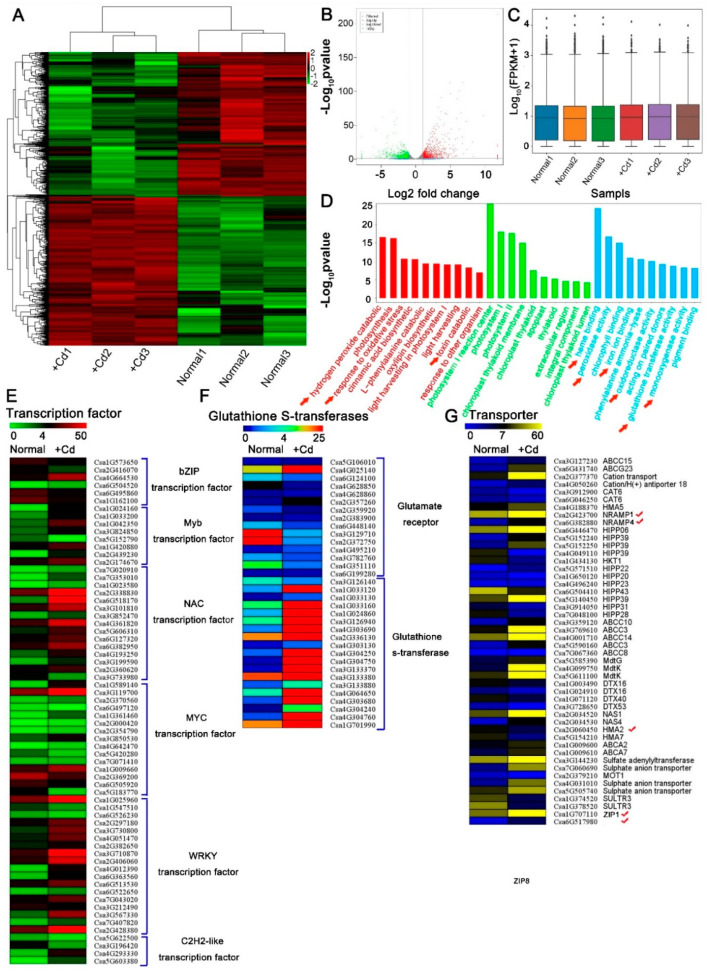
Analysis of Cd-exposed cucumber transcriptome. (**A**) Heatmap representation of a one-dimensional hierarchical clustering of differential gene expression as determined by mRNA-seq for Cd-exposed cucumber relative to normal plant (Cd-free). (**B**) Differential transcript abundance of Cd-free and Cd-treated seedlings. The *x*-axis represents the log2 fold change under the mean normalized expression of all transcripts (*y*-axis). Green (downregulation) and red (upregulation) dots indicate differential genes (*p* < 0.01). (**C**) Box–whisker plot FPKM (fragments per kilobase of exon per million fragments mapped) of six samples (Normal1, Normal2, Normal3, +Cd1, +Cd2 and +Cd3). (**D**) GO enrichment analysis of Cd-exposed and normal cucumber transcripts in seedlings. (**E**–**G**) Hierarchical clustering of differentially expressed mRNAs that were significantly different in transcript abundance between Cd-free and Cd-exposed cucumbers. Heat map represented the gene expression level of Cd-respond mRNAs (*p* < 0.05). √ mean candidate cadmium transporter. Ten-day-old cucumber seedlings were exposed to 0 (Normal) or 50 μM Cd (+Cd) for 4d, and the whole plant was selected for RNA extraction. Significance of differences between the treatments was statistically evaluated (*p* < 0.05).

To identify genes that were differentially expressed under Cd stress, we performed a genome-wide analysis of transcripts using high-throughput RNA-Seq technology. Transcript abundance was assessed in Cd-treated (+Cd) and Cd-free (Normal) cucumber seedlings. We generated 252.47 Gb clean reads with average base quality (Q30) of 94.17% (Supplementary Table S1) and sample correlation coefficients of 87.2–100% (Supplemental Figure S1A,B). A total of 1904 differentially expressed genes (DEGs) under Cd stress were identified (Figure 2A). Cd induced a wide change in gene expression (Figure 2B,C). Compared with control, more genes were positively induced in cucumber with Cd (Figure 2B,C). DEGs were further analyzed by gene ontology (GO) (Figure 2D). Most of the genes were involved in terms including Cd transcriptional responses and transport, hormone and oxidative stress reaction binding, detoxication, signaling, or antioxidative responses (Figure 2D–G and Figure S1C,D), which indicating the mechanisms for Cd accumulation and detoxication in cucumber. The transcript levels of seven genes of NRAMPs, ZIPs, and HMAs were found to be affected by Cd (Figure 2G). Among them, two Nramp family genes (*CsNramp1* and *CsNramp4*), two ZIP family genes (*CsZIP1* and *CsZIP8*), and an HMA family gene (*CsHMA5*) were significantly upregulated at the transcriptional level (Figure 2G). *CsHMA2* and *CsHMA7* were repressed in Cd-exposed cucumber seedlings (Figure 2G). These results indicated that these transporters were possibly involved in Cd absorption and transportation.

### 3.3. Bioinformatics Analysis of Cucumber Cd-Responsive Transporters

In previous studies, the function of CsHMA1 was validated by the yeast system [15]; therefore, four other Cd transporters identified were systematically analyzed by bioinformatics. To study the evolutional relationships of Nramp, HMA and ZIP genes among cucumber, *Arabidopsis*, and rice, we respectively collected a data set of 17 putative Nramp protein sequences, 39 putative ZIP protein sequences, and 25 putative HMA protein sequences including 27 from *Arabidopsis*, 31 from rice, and 23 from cucumber for phylogenetic analysis (Supplemental Figures S2–S4). The full-length protein sequences were used for phylogenetic analysis. The closest homolog of OsNramp5 is similar to CsNramp4, which shares 38% identity (Supplemental Figure S2). Using the SOSUI program (http://bp.nuap.nagoya-u.ac.jp/sosui/, 12 July 2021), we predicted that CsNramp1 and CsNramp4 are membrane proteins with 12 transmembrane domains (Supplemental Figures S5 and S6). Phylogenetic analysis also showed that CsZIP1 and CsZIP8, which have nine transmembrane domains (Supplemental Figures S7 and S8), exhibited 74% identity with OsIRT1 (iron regulated transporter1) and OsIRT2 (Supplemental Figure S3). OsIRT1 and OsIRT2 had an influx activity of Cd^2+^ as well as Fe^2+^ in yeasts [43]. Hence, CsZIP1 and CsZIP8 are potentially involved in Cd uptake. Systematic analysis of cucumber HMA family genes showed that CsHMA2 is the most closely related to HMA2 and HMA3 groups in rice and *A. thaliana*. CsHMA1 and CsHMA2 are the same genes of CsHMA3 and CsHMA4 in previous study on Cd detoxification in cucumber (Supplemental Figure S4) [15]. Therefore, we selected CsNramp1, CsNramp4, CsZIP1, CsZIP8, CsHMA1, and CsHMA2 as candidate Cd transporters for downstream analysis.

### 3.4. Heterologous Expression of CsNramp1/4 and CsZIP1/8 Increased Cd Accumulation in Yeast Cells

**Figure 3:**
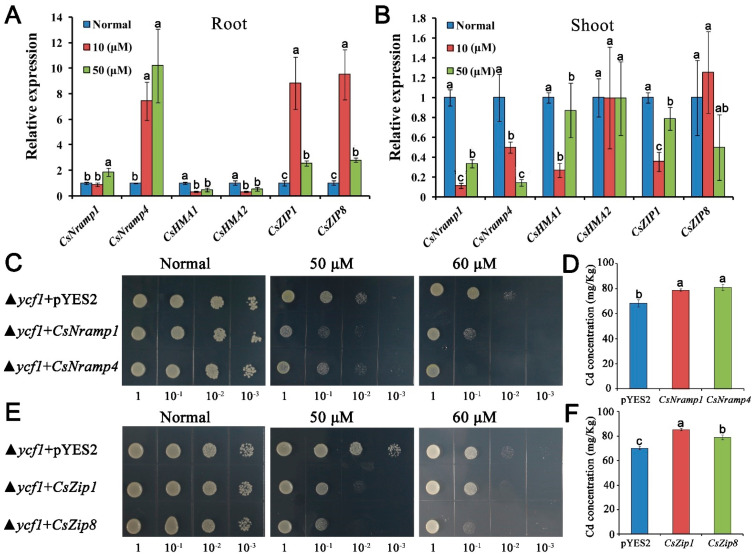
Profiling of gene expression and metal transport capacity of representative genes coding for potential Cd transporter. (**A**,**B**) qRT-PCR analysis of transcript abundance of *CsNramp1*, *CsNramp4*, *CsHMA1*, *CsHMA2*, *CsZIP*, and *CsZIP8* in cucumber root (**A**) and shoot (**B**) under Cd exposure. Ten-day-old seedlings were exposed to 0, 10, or 50 μM Cd (+Cd) for 4 d. Vertical bars represent standard deviation (SD) of the mean of three biological replicates (*n* = 5 seedlings). Different letters on the bars indicate a significant difference (*p* < 0.05) between the treatments. (**C**–**F**) Expression of CsNramp1, CsNramp4, CsZIP1, and CsZIP8 in yeast cells to determine the activity for Cd detoxification. All cell types were grown in YNB medium supplemented with or without 50 or 60 μM Cd for 3 d. (**C**,**E**) Growth response to Cd was determined by comparing transformed and untransformed yeast. D and F: Clones described in the liquid cultures were diluted to OD 0.2 in fresh SD/ura medium, and the Cd concentration in the cells was determined by ICP-MS. Vertical bars represent the standard deviation of three replicates. Different letters on the bars indicate that mean values are significantly different between transformed and untransformed cells (*p* < 0.05).

To examine the transport activity of CsNramp1, CsNramp4, CsZIP1, and CsZIP8 for Cd, we expressed CsNramp1, CsNramp4, CsZIP1, CsZIP8, or pYES2 empty vector in ycf1 yeast strains (a yeast mutant defective in Cd detoxification), respectively. The inhibition of growth was more serious in yeast expressing CsNramp1, CsNramp4, CsZIP1, and CsZIP8 than that expressing pYES2 empty vector (Figure 3C–E). The Cd accumulation in yeast cells was also compared under the control of Gal-inducible promoter using liquid culture. In the presence of Gal, yeast expressing CsNramp1, CsNramp4, CsZIP1, and CsZIP8 significantly accumulated higher Cd compared with the control (Figure 3D–F). These findings indicated that CsNramp1, CsNramp4, CsZIP1, and CsZIP8 are able to transport Cd^2+^ in yeast.

### 3.5. Association Analysis of Ionomics and Gene Expression Profiles Identified the Major Cd Accumulation Genes in Cucumber

**Figure 4:**
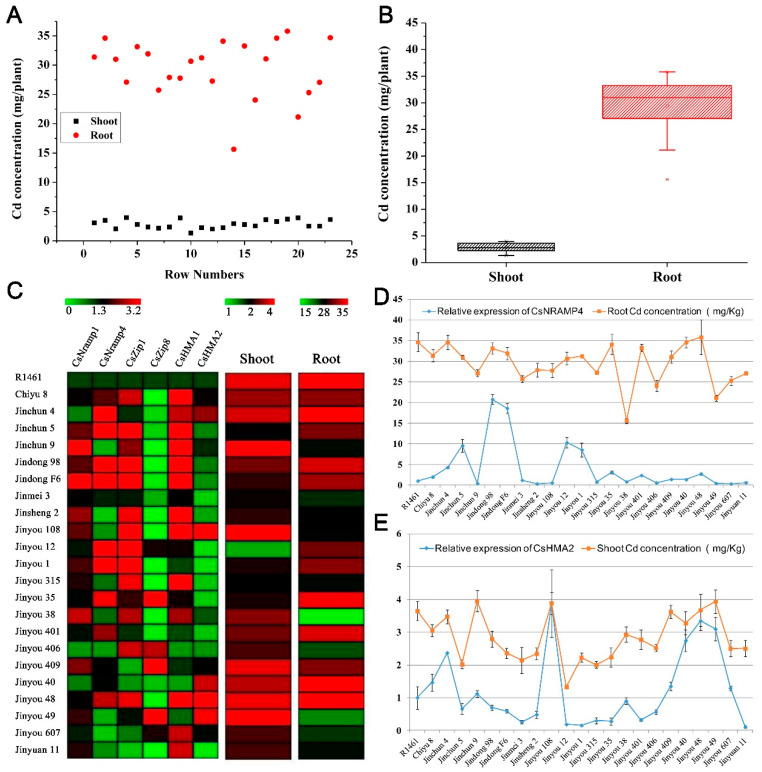
Correlation analysis of ionomics and gene expression profiles identified the major Cd accumulation genes in cucumber. (**A**) Distribution of Cd accumulation in the roots and shoots of 23 cultivated cucumber. The red circle represents shoots, and the black box represents roots. (**B**) Box–whisker plot Cd concentration of all 23 cultivated varieties identified by ICP-MS. (**C**) Hierarchical clustering of six Cd-related transporters expression and Cd accumulation in the shoots and roots of 23 cultivated varieties. Heat map represents the gene transcript level and Cd accumulation. (**D**,**E**) Association analysis of the expression of *CsNramp4* (**D**) and *CsHMA2* (**E**) in 23 cultivated varieties along with Cd content in the roots and shoots. Ten-day-old cucumber plants were grown in nutrient solution supplemented with 0 and 0.5 μM Cd for 10 days.

To explore the association in cucumber between Cd accumulation and the transporters mentioned above, we selected 23 cucumber cultivars collected throughout China and cultured them in soils with simulated Cd pollution conditions (0.5 μM) in facilities. We measured the Cd content in shoots and roots. The Cd accumulation in the roots of all varieties was significantly higher than that in the shoots (Figure 4A,B). At the same time, we also conducted q-PCR detection on the expression levels of the five candidate transporter genes (*CsNramp1*, *CsNramp4*, *CsZIP1*, *CsZIP8*, and *CsHMA2*) and HMA1 [15] in the roots of each variety. The heat map results showed that the six genes were significantly differentially expressed in each variety (Figure 4C). The association analysis showed that the expression of CsNramp4 in the roots was positively correlated with the content of Cd in the roots (Figure 4D). Similarly, the expression of CsHMA2 was positively correlated with the Cd content in the shoots (Figure 4E), indicating that the shoot Cd content in cucumber may be related to CsHMA2 transportation. However, the gene expression levels of *CsNramp1*, *CsZIP1*, *CsZIP8*, and *CsHMA1* were not significantly correlated with Cd content in the roots or shoots, indicating that the four genes were not the major regulatory genes involved in Cd distribution in the roots or shoots of these varieties.

### 3.6. Association Analysis of Cucumber Cd-Related Transporter Gene Expression Profiles and Cd-Induced REDOX Reaction

**Figure 5:**
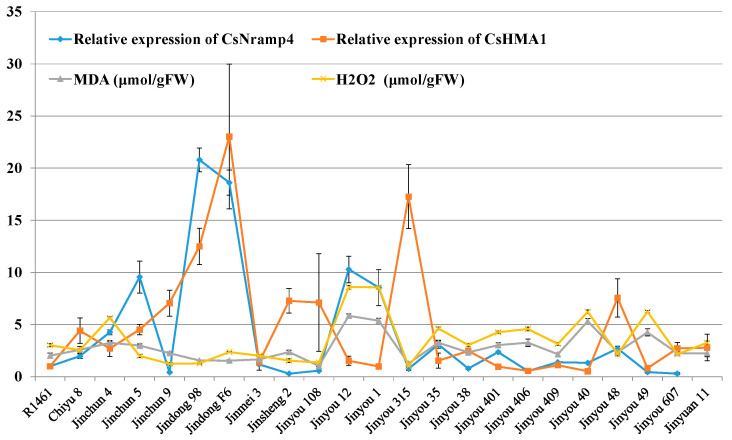
Association analysis of cucumber Cd-related transporter gene expression profiles and Cd-induced REDOX reaction. Correlation analysis of the expression of *CsNramp4* and *CsHMA1* in 23 cultivated varieties along with MDA and H_2_O_2_ content in cucumber seedlings. Ten-day-old cucumber plants were grown in Yamazaki nutrient solution supplemented with 0 and 0.5 μM Cd for 48 h and 4 days.

Our results showed that Cd accumulation in cucumber seedlings was positively correlated with oxidative stress indicators (Figure 1E,F), suggesting that the expression of Cd transporters might assist in the maintenance of REDOX reaction. To verify whether Cd transporters are related to the REDOX reaction induced by Cd stress in cucumber, we quantified the oxidative stress indices of the above 23 cucumber cultivars under Cd stress. Similarly, MDA and H_2_O_2_ were significantly enhanced in 23 varieties (Figure 5). The results of association analysis showed that the expression of CsNramp4 in the roots was positively correlated with oxidative stress indices in the roots (Figure 5). By contrast, the expression of CsHMA1 was negatively correlated with oxidative stress indicators in the roots (Figure 5), suggesting that CsNramp4 and CsHMA1 may maintain the REDOX balance at cytoplasmic and vacuolar levels in cucumber. The gene expression levels of *CsNramp1*, *CsZIP1*, *CsZIP8*, and *CsHMA2* were not directly related to oxidative stress induced by Cd stress in cucumber (Supplemental Figure S9).

### 3.7. Expression Pattern and Subcellular Localization of Cucumber CsNramp1 and 4

In a previous work, the immunostaining with specific antibodies against cucumber proteins revealed tonoplast localization of CsHMA1 and plasma membrane localization of CsHMA2 in cucumber root cells [15]. Here, subcellular localizations of CsNramp1 and CsNramp4 were identified using a construct harboring CsNramp1/4-GFP driven by 35S and introduced into *A. thaliana* protoplasts [24]. CsNramp1-GFP and CsNramp4-GFP proteins were expressed in the cell membrane (Supplemental Figure S10C,D), suggesting that CsNramp1/4 possibly functions in the plasma membrane. The expression of *CsNramp1* and *CsNramp4* was investigated in different tissues at different growth stages. At all growth stages, *CsNramp4* was mainly expressed in the roots (Supplemental Figure S10A). However, *CsNramp1* was mainly expressed in cotyledons and stems (Supplemental Figure S9B). These results indicated that CsNramp4 was involved in Cd absorption at the root cell membrane level. This result can explain that the expression of CsNramp4, but not CsNramp1, in cultivated varieties was positively correlated with Cd content in the roots.

### 3.8. CsNramp4 Conferred High Absorption of Cd and CsHMA2 Responsible for Cd Trans Duction from Root to Shoot

**Figure 6:**
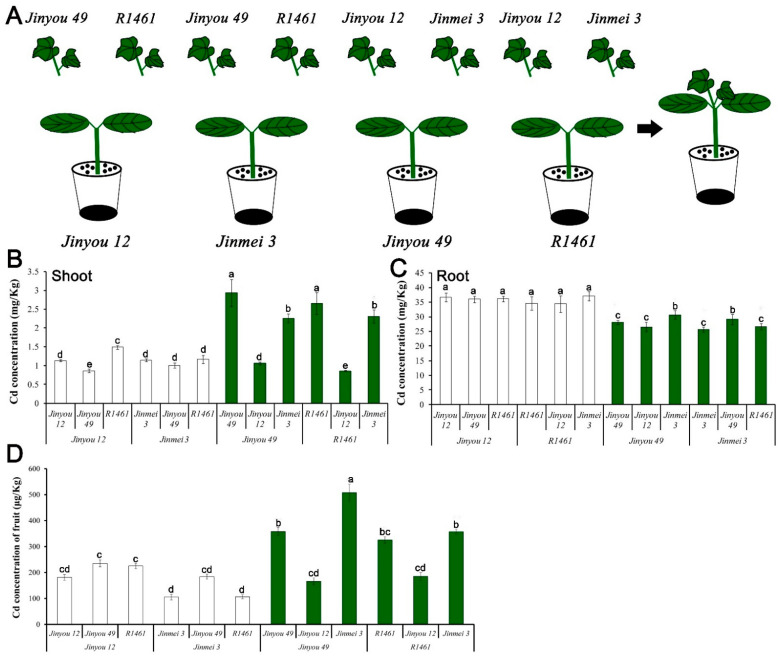
Cadmium accumulation in the roots, shoots, and fruits of cucumber with different grafting combinations exposed to Cd stress. (**A**) Diagram of cucumber grafting combination. (**B**–**D**) Distribution of Cd content in the shoots (**B**), roots (**C**), and fruits (**D**) of cucumber with different graft combinations under 0.5 μM Cd treatment. Ten-day-old cucumber plants were selected for grafting. After 7 days of growth in the vermiculite surrounded by Yamazaki nutrient solution, the live seedlings were supplemented with 0 and 0.5 μM Cd for 48 h and 4day. Vertical bars represent the standard deviation of three replicates. Different letters on the bars indicate that the mean values are significantly different between Jinyou12 lines and other combinations (*p* < 0.05).

Up to now, transgenetic cucumber and mutants were obtained difficultly. To investigate the physiological role of CsNramp4 and CsHMA2 in plants, we designed a series of grafting experiments to verify their function (Figure 6A). We selected four widely cultivated cucumber varieties, which showed significant differential expression of CsNramp4 and CsHMA2 in the roots (Figure 6C). Within 10 d of Cd treatment, we determined the Cd accumulation in cucumber plants. The result indicated that root tissues with high expression of CsHMA2 (*Jinyou49* and *R1461*) accumulated more Cd in shoots, while roots with high level of CsNramp4 (*Jinyou12* and *R1461*) had positive correlation with Cd content in the roots. In rice, the CsNramp4 homologous gene OsNramp5 is localized to the plasma membrane of root cells and functions as a high-affinity transporter for Cd and Mn uptake [44]; it actively mediates the Cd uptake and translocation in rice. CsNramp4 has the same organizational expression pattern with OsNramp5, which has high expression in the roots (Supplemental Figure S10C) [44]. These results indicated that CsNramp4 is mainly responsible for the absorption of Cd by the roots in cucumber. In a previous work, CsHMA2 (i.e., CsHMA4) confers yeast tolerance to Cd and Zn via the enhanced efflux of metals from cells across the plasma membrane [15]. The most closely related genes AtHMA2 and AtHMA4 in *Arabidopsis* displayed a similar Cd efflux function, which are involved in the loading of Cd into the xylem from root-to-shoot transport of Cd [45,46]. Meanwhile, we also detected the content of Cd in the cucumber fruits of the above grafting combination, and the content of Cd in the fruits was basically consistent with that in the shoots (Figure 6D). The content of CsHMA2 in the rootstock determined the accumulation of Cd content in the fruits. Therefore, the high expression of CsHMA2 in cucumber cultivated varieties directly led to the accumulation of Cd in the shoots and fruits.

### 3.9. CsHMA1 Was Positively Correlated with Root Antioxidant Capacity of Cucumber

**Figure 7:**
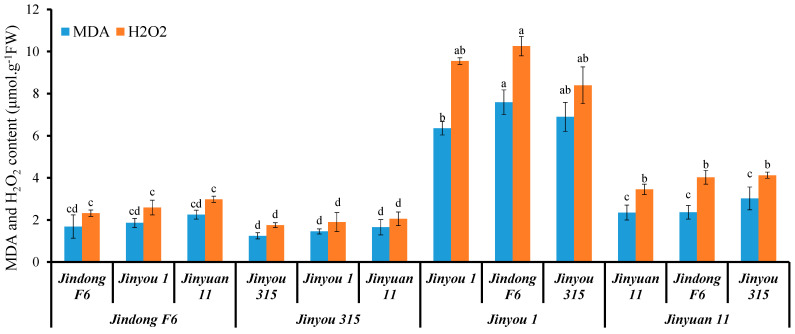
Oxidative stress level in cucumber seedlings with different grafting combinations exposed Cd stress. MDA and H_2_O_2_ content in seedlings of cucumber with different graft combinations under 0.5 μM Cd treatment. Ten-day-old cucumber plants were selected for grafting. After 7 days of growth in the vermiculite surrounded by Yamazaki nutrient solution, the live seedlings were supplemented with 0 and 0.5 μM Cd for 48 h and 4 days. Different letters on the bars indicate that the mean values are significantly different between Jinyou12 lines and other combinations (*p* < 0.05).

We further used four cucumber variety to study whether a direct correlation exists between oxidative stress level and expression of *CsHMA1* and *CsNramp4* in cucumber. Concretely, varieties *Jinyou 1* and *Jinyuan 11* were low in expression of *CsHMA1* in the roots, while Jindong F6 and Jinyou 315 were high in expression of *CsHMA1* as rootstock and scion. In addition, the expression of *CsNramp4* in the roots of *Jinyou 315* and *Jinyuan 11* was lower, while the expression of *CsNramp4* in the roots of *Jindong F6* and *Jinyou 1* was higher. Then, these four cucumber varieties were applied to graft experiments. When the varieties *Jindong F6* and *Jinyou 315* with high *CsHMA1* expression were used as rootstocks, the ROS content in the grafted seedlings was at a relatively low level (Figure 7). However, when *Jinyou 1* and *Jinyuan 11* with lower *CsHMA1* expression were used as rootstocks, the plants of each combination accumulated more MDA and H_2_O_2_ (Figure 7). These results indicated that *CsHMA1* expression level in cucumber roots directly determined Cd-induced ROS content. Although previous results showed that the expression of CsNramp4 was positively correlated with the accumulation of Cd-induced ROS in the plant, when *CsHMA1* was at high expression level, the increased level of CsNramp4 could not increase the accumulation of ROS, indicating that CsHMA1 had a stronger regulation ability of Cd-induced ROS than CsNramp4. This reason may be that Cd absorbed by CsNramp4 in the root is rapidly chelated into vacuoles by CsHMA1, thereby reducing oxidative damages (Figure 7). In conclusion, our results indicated that CsNramp4 is involved in oxidative damages caused by the accumulation of Cd in the root cytoplasm, while CsHMA2 rapidly detoxifies at the root vacuole level to reduce the oxidative stress damage when cucumber grows in Cd-polluted soil.

### 3.10. Overexpression of CsHMA1 Improved Antioxidation Capacity in Plants

**Figure 8:**
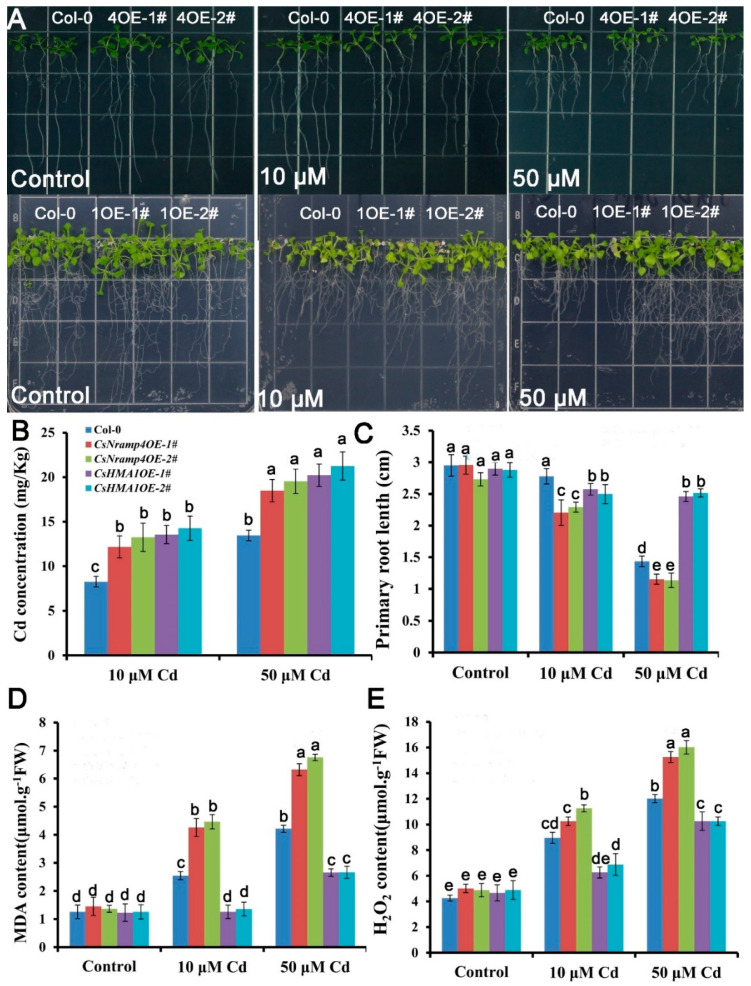
Analysis of Cd tolerance in the wild type (WT), CsNramp4 transgenic lines (*UBI10:CsNramp4*), and CsHMA1 transgenic lines (*UBI10:CsHMA1*) of *Arabidopsis.* (**A**) Phenotype of seedlings grown on 1/2 MS medium with or without 10 or 50 μM Cd for 12 d. (**B**) Primary root length of *UBI10:CsNramp4* and *UBI10:CsHMA1* transgenic seedlings grown on 1/2 MS medium with or without10 and 50 μM Cd for 12 d. (**C**) Cd concentrations in the shoots and roots of WT, *UBI10:CsNramp4* and *UBI10:CsHMA1* plants. (**D**,**E**) MDA (**D**) and H_2_O_2_ (**E**) contents of WT, *UBI10:CsNramp4*, and *UBI10:CsHMA1* transgenic seedlings. Seedlings were grown on 1/2 MS medium with or without 10 or 50 μM Cd for 48 h. Vertical bars represent standard deviation. Different letters on the bars indicate that the mean values are significantly different between the transgenic plants and wild-type plants (*p* < 0.05).

To investigate the effects of CsHMA1 and CsNramp4 on plant REDOX response under Cd stress, we constructed transgenic *A. thaliana* overexpressing *CsHMA1* and *CsNramp4*, respectively. The homozygous OE lines showed 10- to 35-fold higher *CsHMA1* and *CsNramp4* transcript levels than the wild type. Seedlings grown on _1/2_MS medium for 7 days were treated with 0, 10, and 80 μM Cd for 10 d. The growth in OE lines of CsHMA1 exhibited more tolerance to Cd stress than the wild type. The primary root length of the OE lines increased by 12.6–19.2% (Figure 8C). By contrast, the overexpression of *CsNramp4* was more sensitive to Cd toxicity, with the primary root length decreased by 19.9–34.6% compared with the wild type (Figure 8B). Following the analyses of growth responses to Cd, we examined the Cd concentrations in the plants. Both the OE lines of *CsHMA1* and *CsNramp4* had higher Cd concentration than the wild type under Cd stress (Figure 8B). To examine whether the transgenic plants have an antioxidative capability, we performed MDA and H_2_O_2_ detection in Cd-exposed seedlings. Compared with WT, *UBI10:CsHMA1* plants exposure to Cd showed a lower level of MDA and H_2_O_2_, while in *UBI10:CsNramp4* roots, the levels of MDA and H_2_O_2_ were relatively higher in Cd stress (Figure 8D,E). These results suggested that the overexpression of CsHMA1 enhances the antioxidant capacity of plants in response to Cd stress, while the high transcript level of CsNramp4 may enhance oxidative damages through the accumulation of Cd.

## 4. Discussion

Cadmium pollution has attracted worldwide attention [21]. Cd toxicity can cause ROS elevation in plants, oxidative damage, lipid peroxidation, and growth inhibition [47]. Increased H_2_O_2_ and MDA production during short-term exposure of cucumber cell cultures to Cd^2+^ has been reported previously [47]. Many studies have revealed that a large number of Cd-responsive genes may be involved in ROS signal transduction [47]. The relevant signal transduction pathways include Ca–calmodulin system, hormones, and mitogen-activated protein kinase (MAPK) phosphorylation cascade, which converge by activating Cd-related ROS genes [34]. However, the complex regulatory mechanisms for the processes remain largely unknown. In our work, the levels of H_2_O_2_ and MDA were gradually elevated with increasing Cd content in cucumber. The control of Cd accumulation is maintained by a group of metal transporters [7]. Accumulation of heavy metal Cd in plant cells is associated with increased expression of some metal transporters, such as HMAs (heavy metal ATPases) and ZIP family members involved in Cd [15,20]. This study functionally identified some putative locus-encoding Cd transporters that can uptake or chelate Cd in cucumber tissues and cells.

We used transcriptome analysis to screen seven *NRAMPs*, *ZIPs*, and *HMAs* family trans porters in response to cadmium exposure. Our results of gene expression profiles in cucumber tissues showed that *CsNramp4* was highly expressed mainly in cucumber roots, was located in the plasma membrane of cucumber cells, and had similar expression pattern to the main Cd absorption transporter Os*Nramp5* in rice [41,44]. This finding suggests that CsNramp4 plays a key role in Cd absorption in cucumber. At present, ZIP family transporter AtIRT1 has been identified as the main Cd absorber in *A. thaliana*, which is also involved in the absorption of Fe^2+^, Zn^2+^, and Mn^2+^. OsIRT1 is also involved in the absorption of Cd in rice. We also identified CsZIP1 and CsZIP8 as homologous genes of OsIRT1, complementing CsNramp1, CsNramp4, CsZIP1, and CsZIP8 through yeast experiments. It is worth exploring which one above is mainly responsible for the accumulation of Cd ions in cucumber cultivars

In addition, we found that the pattern about CsHMA2 gene in response to Cd stress is the most closely related to HMA2 and HMA4 in plant root transport of Cd to shoot [7,16], suggesting that it may participate in the loading of Cd to the above-ground part. Therefore, we used ionomics and gene expression profiles to analyze the relationship between these transporters and the distribution of Cd ions in the roots and aboveground parts of 23 cucumber cultivars. This comprehensive analysis showed that Cd accumulation in cucumber roots was positively correlated with the expression of *CsNramp4*, while Cd accumulation in cucumber aboveground parts was positively correlated with the expression of *CsHMA2*, suggesting the role of CsNramp4 and CsHMA2 in the absorption and transduction of Cd into cucumber shoots. Grafting experiments further showed that the expression of *CsNramp4* contributed to the accumulation of Cd in cucumber roots, while CsHMA2 was involved in the transport of Cd from the root to shoot. Our work represents the transporter pathway for Cd from soil to the fruit of cucumber.

A large number of Cd accumulation and detoxification transporters have been identified in plants, but the direct relationship between these transporters and Cd-induced oxidative stress is rarely reported [8,9,13,16,44,46,48,49,50,51,52,53,54,55,56]. Our previous research found the overexpression of the heavy metal-associated isoprenylated plant protein (HIPP) subfamily member OsHIPP42 involved in rice tolerance to Cd by reducing the electrolyte leakage and death of cells under Cd stress [23]. The results suggest that Cd transporters may be involved in the regulation of oxidative stress levels in plants. Our results showed that the expression of CsNramp4 in the roots was positively correlated with oxidative stress indices in the roots. Meanwhile, the transcript of *CsHMA1* was negatively correlated with oxidative stress indicators in the roots, suggesting that CsNramp4 and CsHMA1 may maintain the REDOX balance at cytoplasmic and vacuolar levels in cucumber. We further selected cucumber seedlings with different expression levels of CsHMA1 and CsNramp4 as scions and grafting stock to study whether a direct correlation exists between oxidative stress level and expression of CsHMA1 and CsNramp4 in cucumber. The results indicated that the high expression of CsNramp4 aggravated Cd-induced oxidative damage in cucumber roots. The CsHMA1 expression level in cucumber roots directly determined the Cd-induced ROS content. Overexpressed CsNramp4 and CsHMA1 in *Arabidopsis* showed the same ROS change level with cucumber when the seedlings were exposed to Cd. However, when *CsHMA1* was at high expression level, the increased level of CsNramp4 could not increase the accumulation of ROS, indicating that CsHMA1 had a stronger regulation of Cd-induced ROS than CsNramp4. This finding may be because Cd absorbed by CsNramp4 in the root is rapidly chelated into vacuoles by CsHMA1, thereby reducing oxidative damage. In *Arabidopsis*, seedlings employ a two-step mechanism to detoxify toxic ions. First, phytochelatins, such as GSH and PCs, bind to the toxic ion. Then, the metal–phytochelatin complex is sequestered by two ABCC-type transporters, AtABCC1 and AtABCC2, in the vacuole [57]. Effective GSH and PCs can rapidly reduce Cd-induced ROS and enhanced Cd tolerance and accumulation. This chelation event is followed by the transport of PC–Cd complexes into the vacuole, which is catalyzed by the ABC transporter. Unlike ABC transporters, the plant HMA3 genes can directly bind to Cd ions and rapidly chelate toxic Cd from the cytoplasm into vacuoles, thus achieving detoxification. CsHMA1 seems to have either phytochelatin binding or ABC transporter isolation abilities in cucumber root cells. These transporters provide useful tools for genetic engineering of plants with enhanced metal tolerance and accumulation, which are desirable characteristics for phytoremediation.

Our study is a good example of a molecular approach for mining of Cd accumulation transporters in greenhouse vegetables. Root uptake and chelating, xylem loading, and phloem transportation are important transport processes that determine Cd accumulation in the edible parts of crops [17,44]. In the molecular design breeding, a possible strategy is the marker-assisted breeding. For example, identification of a QTL associated with CsHMA2 expression in vegetable crop for low-Cd accumulation in aboveground edible part from a low-Cd accumulating cultivar cucumber. QTLs associated with high expression of CsHMA1 confer plants with the potential of high Cd resistance. Another possible practical approach is to screen non-functional allele of CsHMA2 from physically or chemically mutagenized populations with a background of major cultivars. In future breeding, gene editing can be used to modify the expression of multi-site transporters to balance Cd accumulation in fruits and the high antioxidant capacity in roots. Our work will provide a useful basis for the development of alternative strategies to genetically engineer low-Cd content and high-antioxidant capacity cucurbit crops to improve green crop production and ensure food safety. Furthermore, future research will provide genetic evidence about regulating Cd-induced ROS in higher plants.

## 5. Conclusions

This study demonstrated that five Cd-responsive transporters transcriptionally responded to Cd stress. Functional identification revealed that CsNramp4 and CsHMA2 led to high accumulation of Cd in the cucumber roots and shoots, respectively. Importantly, high CsHMA1 expression resulted in cucumber tolerance to Cd stress by strengthening antioxidant capacity. Grafting evidence showed that the reduced expression of *CsHMA2* in stocks was found to be associated with low Cd accumulation in the fruits, which contributed to the cucumber accumulation of less Cd in plants. Our work not only helps to understand the transport and regulatory mechanisms underlying Cd detoxification and accumulation in greenhouse vegetables, but it also addresses the environmental issues of Cd contamination in the greenhouse.

## Data Availability

The data presented in this study are available in the article and supplementary material.

## Supplementary Materials

The following are available online at https://www.mdpi.com/article/10.3390/antiox10121973/s1, Figure S1: (A) Principal component analysis (PCA) of gene expression in six cucumber transcriptome samples. (B) Heat diagram of correlation coefficient between six cucumber samples. (C,D) Hierarchical clustering of differentially expressed mRNAs that were significantly different in transcript abundance between Cd-free and Cd-exposed cucumber. Heat map represented the gene expression level of Cd-respond hormone related genes (C) and REDOX reaction genes (D). Ten-day-old cucumber seedlings were exposed to 0 (Normal) or 50 μM Cd (+Cd) for 4d. Significance of differences between the treatments was statistically evaluated (*p* < 0.05). Figure S2: Phylogenic tree of Nramp proteins in the plant kingdom. Phylogenetic relationship of Nramp proteins in cucumber (Red triangle), Arabidopsis (Blue circle), and rice (Green box). The scale shows substitution distance. Figure S3. Phylogenic tree of ZIP proteins in the plant kingdom. Phylogenetic relationship of ZIP proteins in cucumber (Red triangle), Arabidopsis (Blue circle), and rice (Green box). The scale shows substitution distance. Figure S4. Phylogenic tree of HMA proteins in the plant kingdom. Phylogenetic relationship of HMA proteins in cucumber (Red triangle), Arabidopsis (Blue circle), and rice (Green box). The scale shows substitution distance. Figure S5. Gene structure of CsNramp1. (A) Gene structure of CsNramp1. Green boxes and gray boxes show exon of coding region and intron, respectively. (B) Transmembrane domains predicted with SOSUI program. Figure S6. Gene structure of CsNramp4. (A) Gene structure of CsNramp4. Green boxes and gray boxes show exon of coding region and intron, respectively. (B) Transmembrane domains predicted with SOSUI program. Figure S7. Gene structure of CsZIP1. (A) Gene structure of CsZIP1. Green, yellow and gray boxes show untranslated region, exon of coding region and intron, respectively. (B) Transmembrane domains predicted with SOSUI program. Figure S8. Gene structure of CsZIP8. (A) Gene structure of CsZIP8. Green boxes showexon of coding region. (B) Transmembrane domains predicted with SOSUI program. Figure S9. Association analysis of cucumber Cd-related transporter genes expression profiles and Cd induced REDOX reaction. Correlation analysis the expression of CsNramp1, CsZIP1, CsZIP8 and CsHMA2 in 23 cultivated varieties along with MDA and H2O2 content in cucumber seedlings. Ten day-old cucumber plants were grown in the vermiculite surround by Yamazaki nutrient solution supplemented with 0 and 0.5 μM Cd for 48 h and 4day. Figure S10. Expression patterns of CsNramp1 and CsNramp4 in wild-type (WT, R1461) under normal conditions. (A,B) Relative expression in various tissues at different growth stages. Cucumber was grown in a flowerpots until ripening and tissues were sampled. The expression level was determined by quantitative RT-PCR. Vertical bars represent standard deviation of biological triplicate. (C,D) Subcellular localization of CsNramp1 and CsNramp4 protein in Arabidopsis thaliana plants by confocal images. The CsNramp1/4-GFP fusion was constructed and transiently expressed in Arabidopsis protoplasts. C: CsNramp1-GFP. D: CsNramp4-GFP. Table S1: Output data of RNA-seq from four cucumber libraries exposed −Cd and +Cd.

## References

[1] Xu L., Lu A., Wang J., Ma Z., Pan L., Feng X., Luan Y. (2015). Accumulation status, sources and phytoavailability of metals in greenhouse vegetable production systems in Beijing, China. Ecotoxicol. Environ. Saf..

[2] Zhou J., Xia X.J., Hu Z.J., Fan P.X., Shi K., Zhou Y.H., Yu J.Q. (2021). Technological Development and Production of Protected Vegetable in China During‘The Thirteenth Five-year Plan’and Future Prospect. China Veg..

[3] Yang L., Huang B., Hu W., Chen Y., Mao M., Yao L. (2014). The impact of greenhouse vegetable farming duration and soil types on phytoavailability of heavy metals and their health risk in eastern China. Chemosphere.

[4] Hu W., Huang B., Tian K., Holm P.E., Zhang Y. (2017). Heavy metals in intensive greenhouse vegetable production systems along Yellow Sea of China: Levels, transfer and health risk. Chemosphere.

[5] Hu W., Huang B., Borggaard O.K., Ye M., Tian K., Zhang H., Holm P.E. (2018). Soil threshold values for cadmium based on paired soil-vegetable content analyses of greenhouse vegetable production systems in China: Implications for safe food production. Env. Pollut..

[6] Feng S., Zhang J., Mu Z., Wang Y., Wen C., Wu T., Yu C., Li Z., Wang H. (2020). Recent progress on the molecular breeding of *Cucumis sativus* L. in China. Theor. Appl. Genet..

[7] Clemens S., Aarts M.G., Thomine S., Verbruggen N. (2013). Plant science: The key to preventing slow cadmium poisoning. Trends Plant Sci..

[8] Connolly E.L., Guerinot F. (2002). Expression of the IRT1 Metal Transporter Is Controlled by Metals at the Levels of Transcript and Protein Accumulation. Plant Cell.

[9] Vert G., Grotz N., Dedaldechamp F., Gaymard F., Guerinot M.L., Briat J.F., Curie C. (2002). IRT1, an Arabidopsis transporter essential for iron uptake from the soil and for plant growth. Plant Cell.

[10] Zhang H., Hu Z., Lei C., Zheng C., Wang J., Shao S., Li X., Xia X., Cai X., Zhou J. (2018). A Plant Phytosulfokine Peptide Initiates Auxin-Dependent Immunity through Cytosolic Ca(2+) Signaling in Tomato. Plant Cell.

[11] Uraguchi S., Mori S., Kuramata M., Kawasaki A., Arao T., Ishikawa S. (2009). Root-to-shoot Cd translocation via the xylem is the major process determining shoot and grain cadmium accumulation in rice. J. Exp. Bot..

[12] Miyadate H., Adachi S., Hiraizumi A., Tezuka K., Nakazawa N., Kawamoto T., Katou K., Kodama I., Sakurai K., Takahashi H. (2011). OsHMA3, a P1B-type of ATPase affects root-to-shoot cadmium translocation in rice by mediating efflux into vacuoles. New Phytol..

[13] Ueno D., Yamaji N., Kono I., Huang C.F., Ando T., Yano M., Ma J.F. (2010). Gene limiting cadmium accumulation in rice. Proc. Natl. Acad. Sci. USA.

[14] Morel M., Crouzet J., Gravot A., Auroy P., Leonhardt N., Vavasseur A., Richaud P. (2009). AtHMA3, a P1B-ATPase allowing Cd/Zn/Co/Pb vacuolar storage in Arabidopsis. Plant Physiol..

[15] Migocka M., Papierniak A., Maciaszczyk-Dziubinska E., Posyniak E., Kosieradzka A. (2015). Molecular and biochemical properties of two P1B2-ATPases, CsHMA3 and CsHMA4, from cucumber. Plant Cell Environ..

[16] Takahashi R., Ishimaru Y., Shimo H., Ogo Y., Senoura T., Nishizawa N.K., Nakanishi H. (2012). The OsHMA2 transporter is involved in root-to-shoot translocation of Zn and Cd in rice. Plant Cell Environ..

[17] Uraguchi S., Kamiya T., Sakamoto T., Kasai K., Sato Y., Nagamura Y., Yoshida A., Kyozuka J., Ishikawa S., Fujiwara T. (2011). Low-affinity cation transporter (OsLCT1) regulates cadmium transport into rice grains. Proc. Natl. Acad. Sci. USA.

[18] Lee S., Kim Y.Y., Lee Y., An G. (2007). Rice P1B-type heavy-metal ATPase, OsHMA9, is a metal efflux protein. Plant Physiol..

[19] Luo J.S., Huang J., Zeng D.L., Peng J.S., Zhang G.B., Ma H.L., Guan Y., Yi H.Y., Fu Y.L., Han B. (2018). A defensin-like protein drives cadmium efflux and allocation in rice. Nat. Commun..

[20] Liu X.S., Feng S.J., Zhang B.Q., Wang M.Q., Cao H.W., Rono J.K., Chen X., Yang Z.M. (2019). OsZIP1 functions as a metal efflux transporter limiting excess zinc, copper and cadmium accumulation in rice. BMC Plant Biol..

[21] Feng S.J., Liu X.S., Ma L.Y., Khan I.U., Rono J.K., Yang Z.M. (2020). Identification of epigenetic mechanisms in paddy crop associated with lowering environmentally related cadmium risks to food safety. Environ. Pollut..

[22] Zhang B.Q., Liu X.S., Feng S.J., Zhao Y.N., Wang L.L., Rono J.K., Li H., Yang Z.M. (2020). Developing a cadmium resistant rice genotype with OsHIPP29 locus for limiting cadmium accumulation in the paddy crop. Chemosphere.

[23] Khan I.U., Rono J.K., Liu X.S., Feng S.J., Yang Z.M. (2020). Functional characterization of a new metallochaperone for reducing cadmium concentration in rice crop. J. Clean. Prod..

[24] Feng S.J., Liu X.S., Cao H.W., Yang Z.M. (2021). Identification of a rice metallochaperone for cadmium tolerance by an epigenetic mechanism and potential use for clean up in wetland. Environ. Pollut..

[25] Romero-Puertas M.C., Corpas F.J., Rodriguez-Serrano M., Gomez M., Del R.L.A., Sandalio L.M. (2007). Differential expression and regulation of antioxidative enzymes by cadmium in pea plants. J. Plant Physiol..

[26] Dat J., Vandenabeele S., Vranova E., van Montagu M., Inze D., van Breusegem F. (2000). Dual action of the active oxygen species during plant stress responses. Cell Mol. Life Sci..

[27] Hu Z., Li J., Ding S., Cheng F., Li X., Jiang Y., Yu J., Foyer C.H., Shi K. (2021). The protein kinase CPK28 phosphorylates ascorbate peroxidase and enhances thermotolerance in tomato. Plant Physiol..

[28] Ahammed G.J., Li X., Zhang G., Zhang H., Shi J., Pan C., Yu J., Shi K. (2018). Tomato photorespiratory glycolate-oxidase-derived H2 O2 production contributes to basal defence against Pseudomonas syringae. Plant Cell Environ..

[29] Wang H.S., Zhu Z.J., Feng Z., Zhang S.G., Yu C. (2012). Antisense-mediated depletion of GMPase gene expression in tobacco decreases plant tolerance to temperature stresses and alters plant development. Mol. Biol. Rep..

[30] Wang H.S., Yu C., Tang X.F., Zhu Z.J., Ma N.N., Meng Q.W. (2014). A tomato endoplasmic reticulum (ER)-type omega-3 fatty acid desaturase (LeFAD3) functions in early seedling tolerance to salinity stress. Plant Cell Rep..

[31] Cho U., Park J. (2000). Mercury-induced oxidative stress in tomato seedlings. Plant Sci..

[32] Feng S.J., Liu X.S., Tao H., Tan S.K., Chu S.S., Oono Y., Zhang X.D., Chen J., Yang Z.M. (2016). Variation of DNA methylation patterns associated with gene expression in rice (*Oryza sativa*) exposed to cadmium. Plant Cell Environ..

[33] Feng S.J., Zhang X.D., Liu X.S., Tan S.K., Chu S.S., Meng J.G., Zhao K.X., Zheng J.F., Yang Z.M. (2016). Characterization of long non-coding RNAs involved in cadmium toxic response in Brassica napus. RSC Adv..

[34] DalCorso G., Farinati S., Furini A. (2010). Regulatory networks of cadmium stress in plants. Plant Signal Behav..

[35] Anders S., Pyl P.T., Huber W. (2015). HTSeq—A Python framework to work with high-throughput sequencing data. Bioinformatics.

[36] Roberts A., Trapnell C., Donaghey J., Rinn J.L., Pachter L. (2011). Improving RNA-Seq expression estimates by correcting for fragment bias. Genome Biol..

[37] Trapnell C., Williams B.A., Pertea G., Mortazavi A., Kwan G., van Baren M.J., Salzberg S.L., Wold B.J., Pachter L. (2010). Transcript assembly and quantification by RNA-Seq reveals unannotated transcripts and isoform switching during cell differentiation. Nat. Biotechnol..

[38] Kumar S., Stecher G., Tamura K. (2016). MEGA7: Molecular Evolutionary Genetics Analysis Version 7.0 for Bigger Datasets. Mol. Biol. Evol..

[39] Song J., Feng S.J., Chen J., Zhao W.T., Yang Z.M. (2017). A cadmium stress-responsive gene AtFC1 confers plant tolerance to cadmium toxicity. BMC Plant Biol..

[40] Feng S., Xu Y., Guo C., Zheng J., Zhou B., Zhang Y., Ding Y., Zhang L., Zhu Z., Wang H. (2016). Modulation of miR156 to identify traits associated with vegetative phase change in tobacco (Nicotiana tabacum). J. Exp. Bot..

[41] Ishikawa S., Ishimaru Y., Igura M., Kuramata M., Abe T., Senoura T., Hase Y., Arao T., Nishizawa N.K., Nakanishi H. (2012). Ion-beam irradiation, gene identification, and marker-assisted breeding in the development of low-cadmium rice. Proc. Natl. Acad. Sci. USA.

[42] Ding Y., Gong S., Wang Y., Wang F., Bao H., Sun J., Cai C., Yi K., Chen Z., Zhu C. (2018). MicroRNA166 Modulates Cadmium Tolerance and Accumulation in Rice. Plant Physiol..

[43] Nakanishi H., Ogawa I., Ishimaru Y., Mori S., Nishizawa N.K. (2006). Iron deficiency enhances cadmium uptake and translocation mediated by the Fe2+transporters OsIRT1 and OsIRT2 in rice. Soil Sci. Plant Nutr..

[44] Sasaki A., Yamaji N., Yokosho K., Ma J.F. (2012). Nramp5 is a major transporter responsible for manganese and cadmium uptake in rice. Plant Cell.

[45] Haydon M.J., Cobbett C.S. (2007). Transporters of ligands for essential metal ions in plants. New Phytol..

[46] Verret F., Gravot A., Auroy P., Leonhardt N., David P., Nussaume L., Vavasseur A., Richaud P. (2004). Overexpression of AtHMA4 enhances root-to-shoot translocation of zinc and cadmium and plant metal tolerance. FEBS Lett..

[47] Luo S., Tang Z., Yu J., Liao W., Xie J., Lv J., Feng Z., Dawuda M.M. (2020). Hydrogen sulfide negatively regulates cd-induced cell death in cucumber (Cucumis sativus L) root tip cells. BMC Plant Biol..

[48] Thomine S., Wang R., Ward J.M., Crawford N.M., Schroeder J.I. (2000). Cadmium and iron transport by members of a plant metal transporter family in Arabidopsis with homology to Nramp genes. Proc. Natl. Acad. Sci. USA.

[49] Hussain D., Haydon M.J., Wang Y., Wong E., Sherson S.M., Young J., Camakaris J., Harper J.F., Cobbett C.S. (2004). P-type ATPase heavy metal transporters with roles in essential zinc homeostasis in Arabidopsis. Plant Cell.

[50] Lee S., An G. (2009). Over-expression of OsIRT1 leads to increased iron and zinc accumulations in rice. Plant Cell Environ..

[51] Wong C.K.E., Cobbett C.S. (2009). HMA P-type ATPases are the major mechanism for root-to-shoot Cd translocation in Arabidopsis thaliana. New Phytol..

[52] Takahashi R., Ishimaru Y., Senoura T., Shimo H., Ishikawa S., Arao T., Nakanishi H., Nishizawa N.K. (2011). The OsNRAMP1 iron transporter is involved in Cd accumulation in rice. J. Exp. Bot..

[53] Satoh-Nagasawa N., Mori M., Nakazawa N., Kawamoto T., Nagato Y., Sakurai K., Takahashi H., Watanabe A., Akagi H. (2012). Mutations in rice (Oryza sativa) heavy metal ATPase 2 (OsHMA2) restrict the translocation of zinc and cadmium. Plant Cell Physiol..

[54] Yamaji N., Xia J., Mitani-Ueno N., Yokosho K., Ma J.F. (2013). Preferential Delivery of Zinc to Developing Tissues in Rice Is Mediated by P-Type Heavy Metal ATPase OsHMA2. Plant Physiol..

[55] Sasaki A., Yamaji N., Ma J.F. (2014). Overexpression of OsHMA3 enhances Cd tolerance and expression of Zn transporter genes in rice. J. Exp. Bot..

[56] Yan J., Wang P., Wang P., Yang M., Lian X., Tang Z., Huang C.F., Salt D.E., Zhao F.J. (2016). A loss-of-function allele of OsHMA3 associated with high cadmium accumulation in shoots and grain of Japonica rice cultivars. Plant Cell Environ..

[57] Park J., Song W.Y., Ko D., Eom Y., Hansen T.H., Schiller M., Lee T.G., Martinoia E., Lee Y. (2012). The phytochelatin transporters AtABCC1 and AtABCC2 mediate tolerance to cadmium and mercury. Plant J..

