# Supplementary material for: RNA-Seq Identification of Cd Responsive Transporters Provides Insights into the Association of Oxidation Resistance and Cd Accumulation in Cucumis sativus L
Source: Antioxidants (Basel). 2021 Dec 10;10(12):1973. doi: 10.3390/antiox10121973 (PMC8750378; doi:10.3390/antiox10121973)
Supplement: Supplementary file 1 [file antioxidants-10-01973-s001.zip › antioxidants-1446689-supplementary.pdf]

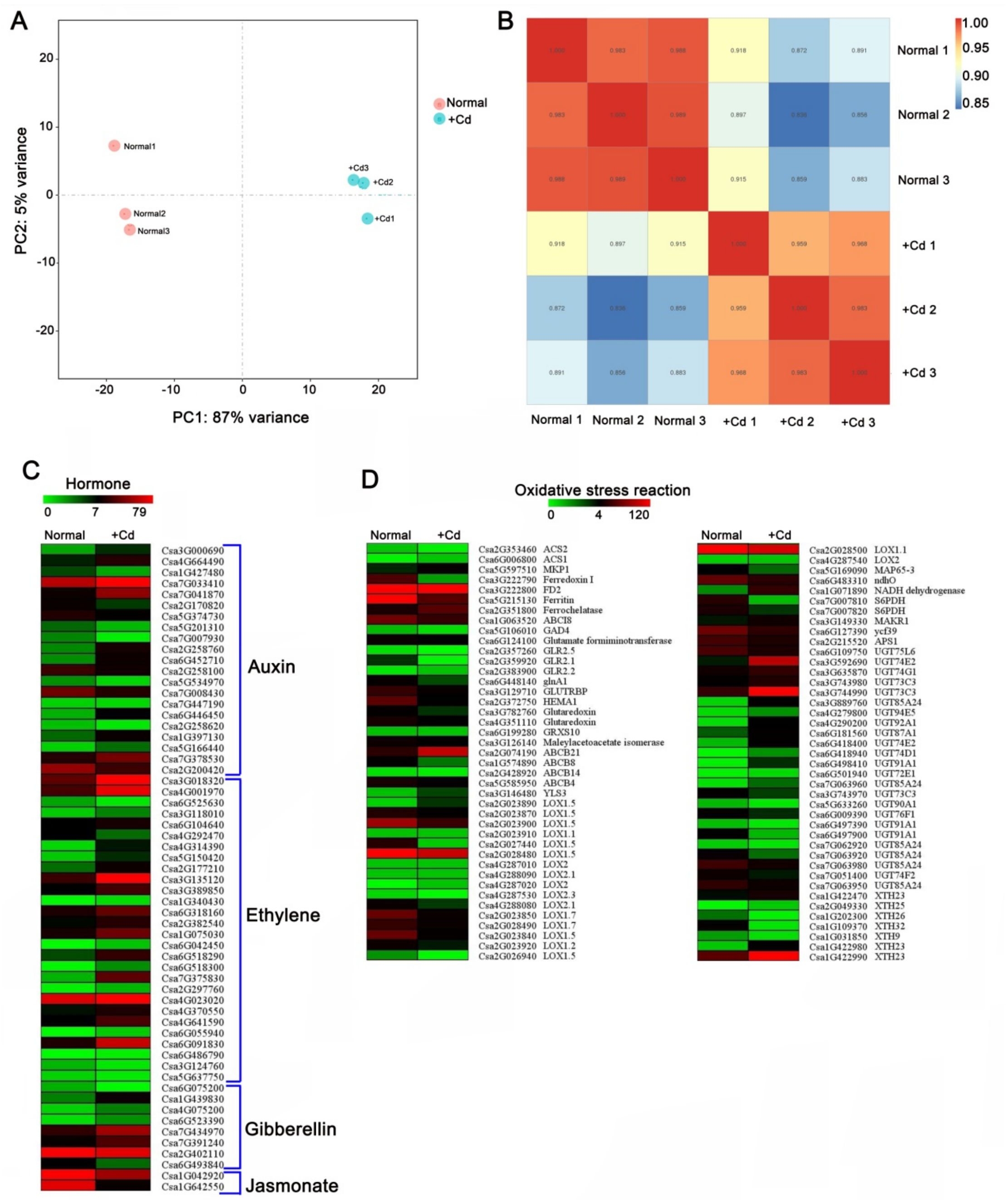

Figure S1. (**A**) Principal component analysis (PCA) of gene expression in six cucumber transcriptome samples.(**B**) Heat diagram of correlation coefficient between six cucumber samples. (**C**,**D**) Hierarchical clustering of differentially expressed mRNAs that were significantly different in transcript abundance between Cd-free and Cd-exposed cucumber. Heat map represented the gene expression level of Cd-respond hormone related genes (**C**) and REDOX reaction genes (**D**). Ten-day-old cucumber seedlings were exposed to 0 (Normal) or 50 μM Cd (+Cd) for 4d. Significance of differences between the treatments was statistically evaluated ($p < 0.05$).

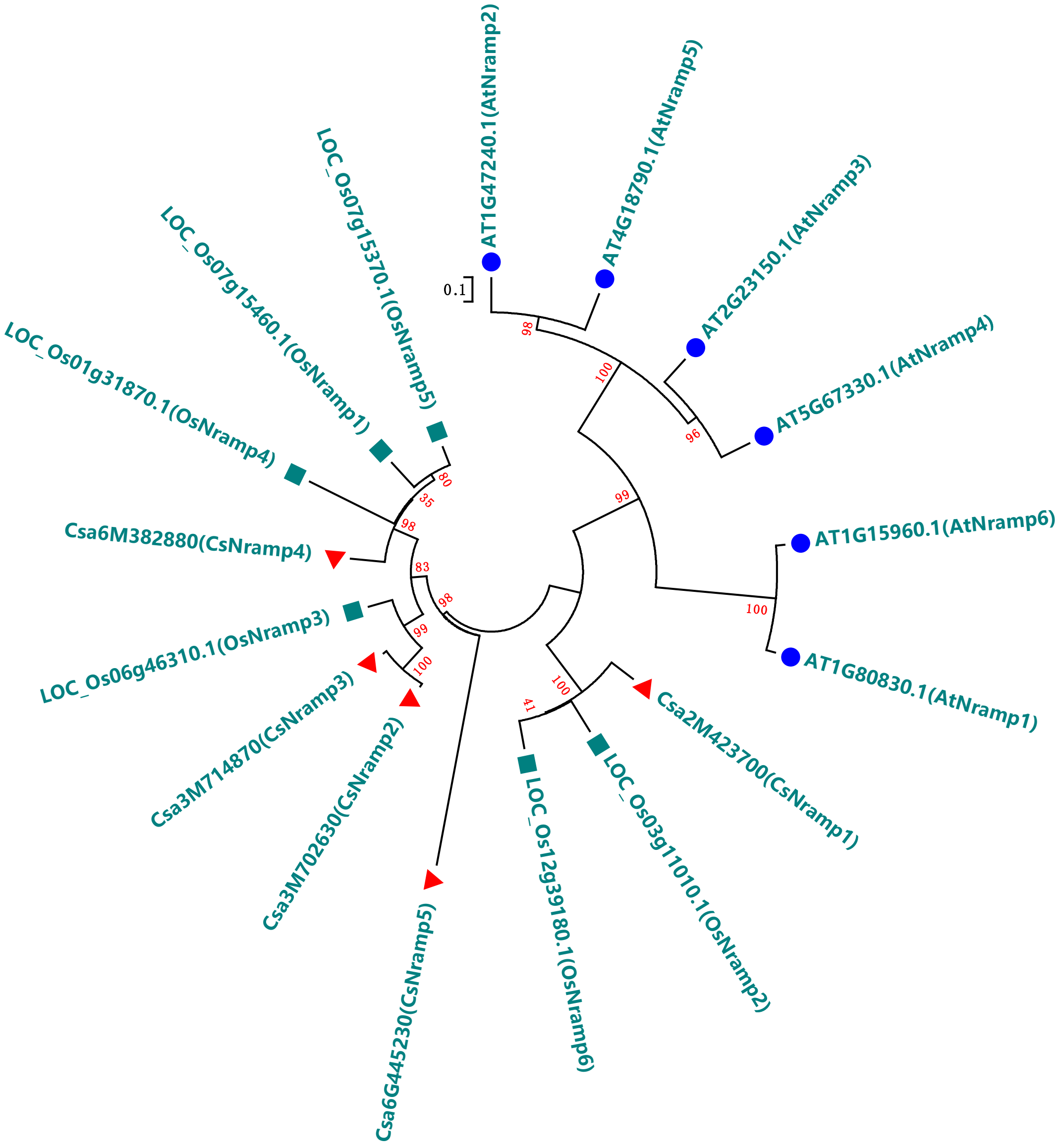

Figure S2. Phylogenic tree of Nramp proteins in the plant kingdom. Phylogenetic relationship of Nramp proteins in cucumber (Red triangle), Arabidopsis (Blue circle), and rice (Green box). The scale shows substitution distance.

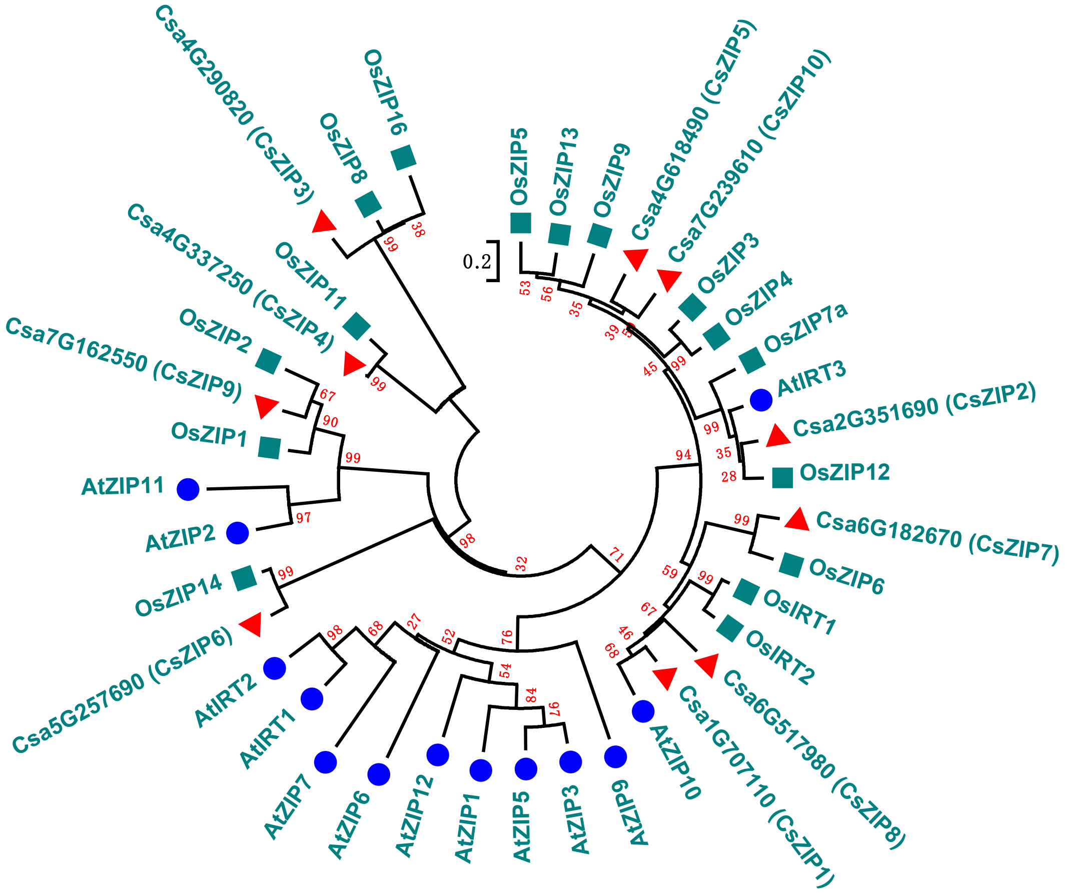

Figure S3. Phylogenic tree of ZIP proteins in the plant kingdom. Phylogenetic relationship of ZIP proteins in cucumber (Red triangle), Arabidopsis (Blue circle), and rice (Green box). The scale shows substitution distance.

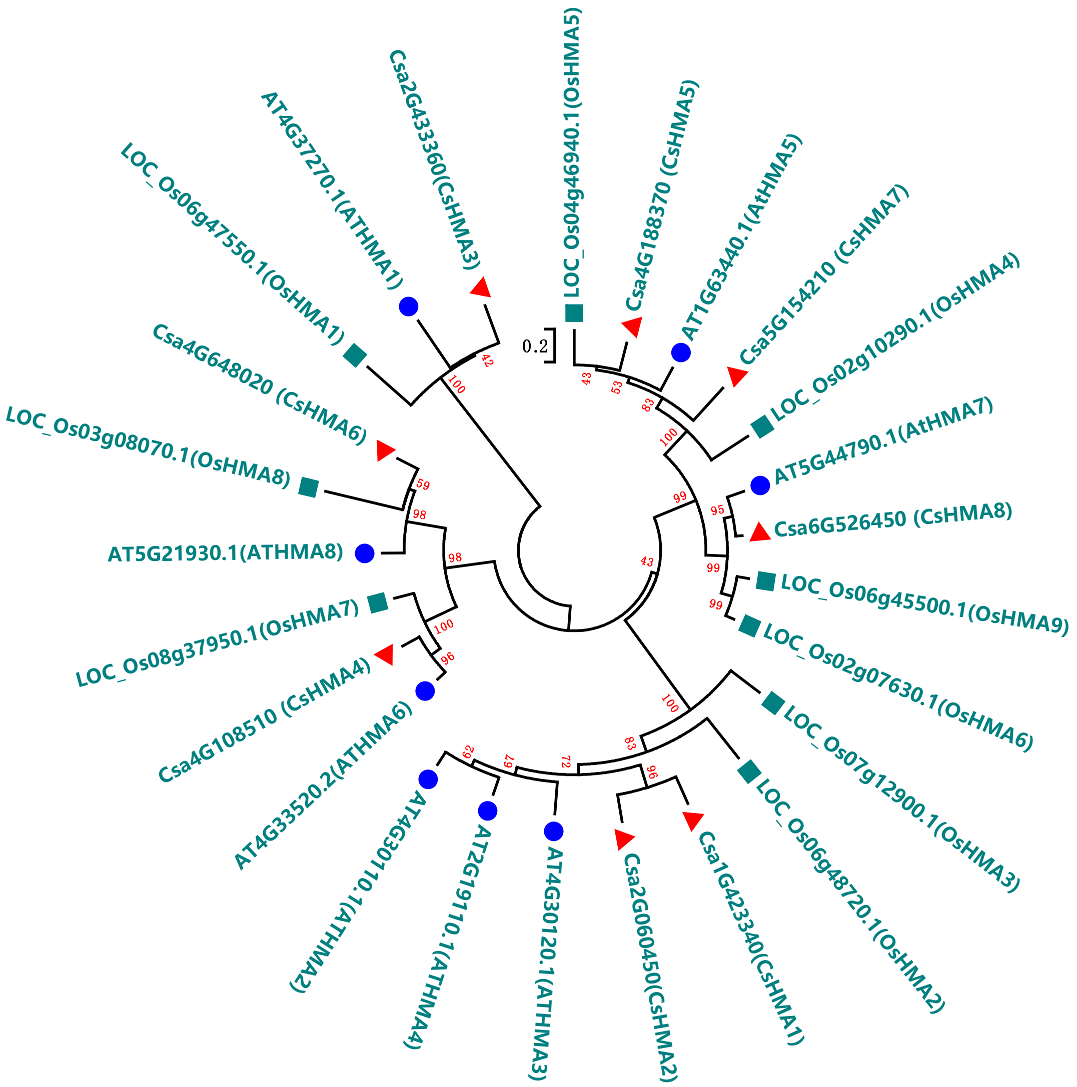

Figure S4. Phylogenic tree of HMA proteins in the plant kingdom. Phylogenetic relationship of HMA proteins in cucumber (Red triangle), Arabidopsis (Blue circle), and rice (Green box). The scale shows substitution distance.

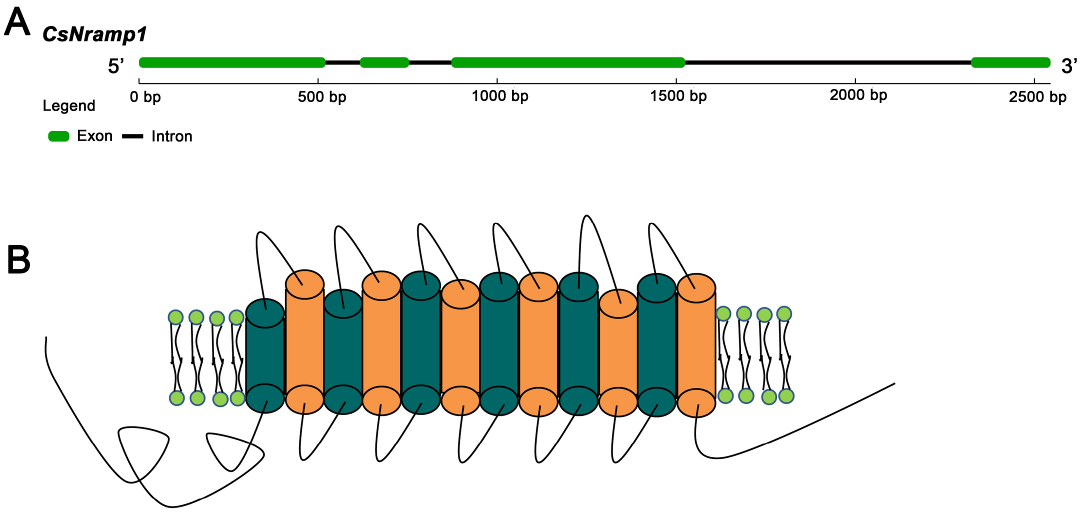

Figure S5. Gene structure of *CsNramp1*. (**A**) Gene structure of *CsNramp1*. Green boxes and gray boxes show exon of coding region and intron, respectively. (**B**) Transmembrane domains predicted with SOSUI program.

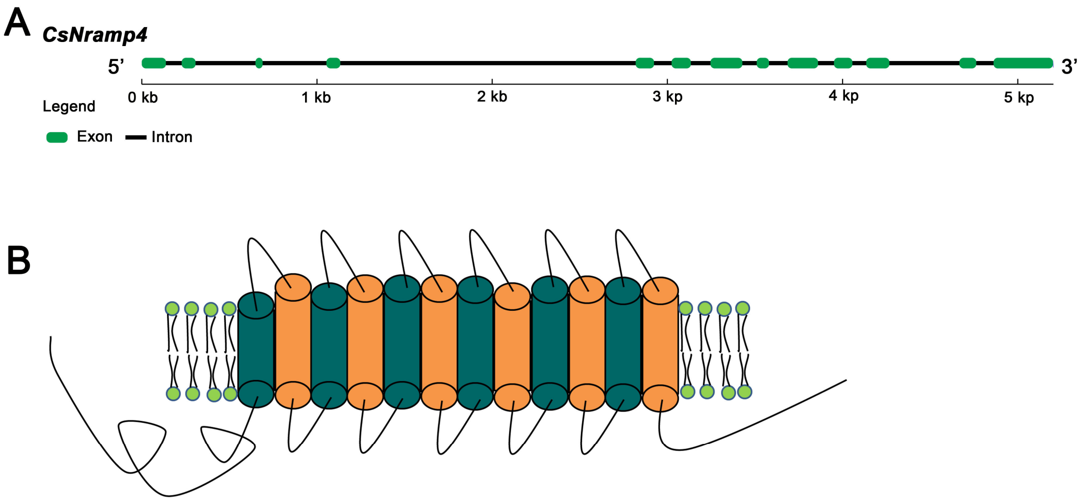

Figure S6. Gene structure of *CsNramp4*. (**A**) Gene structure of *CsNramp4*. Green boxes and gray boxes show exon of coding region and intron, respectively. (**B**) Transmembrane domains predicted with SOSUI program.

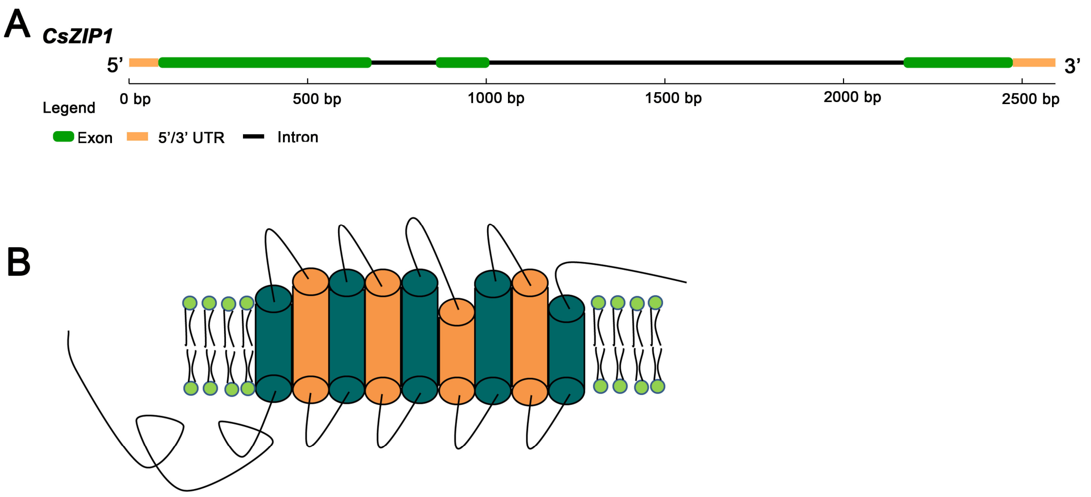

Figure S7. Gene structure of *CsZIP1*. (**A**) Gene structure of *CsZIP1*. Green, yellow and gray boxes show untranslated region, exon of coding region and intron, respectively. (**B**) Transmembrane domains predicted with SOSUI program.

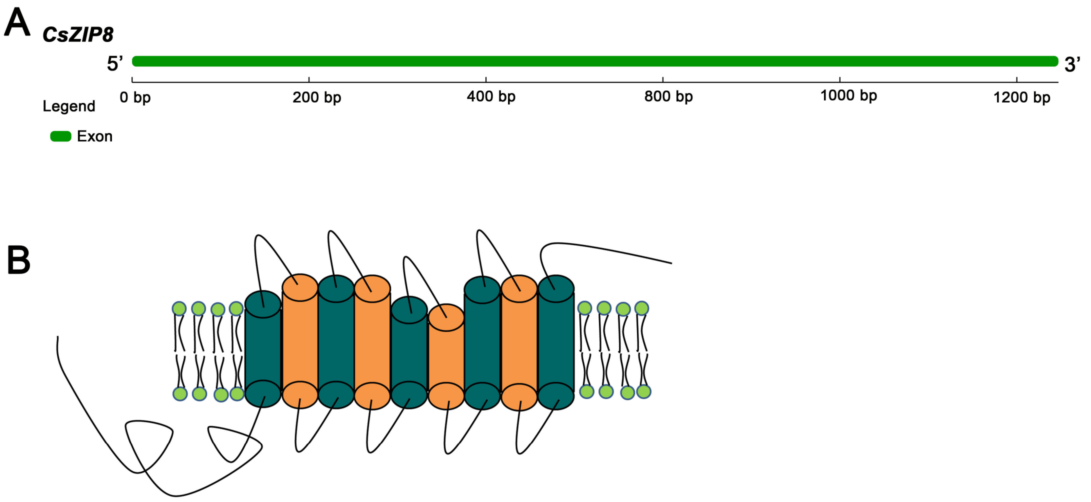

Figure S8. Gene structure of *CsZIP8*. (**A**) Gene structure of *CsZIP8*. Green boxes showexon of coding region. (**B**) Transmembrane domains predicted with SOSUI program.

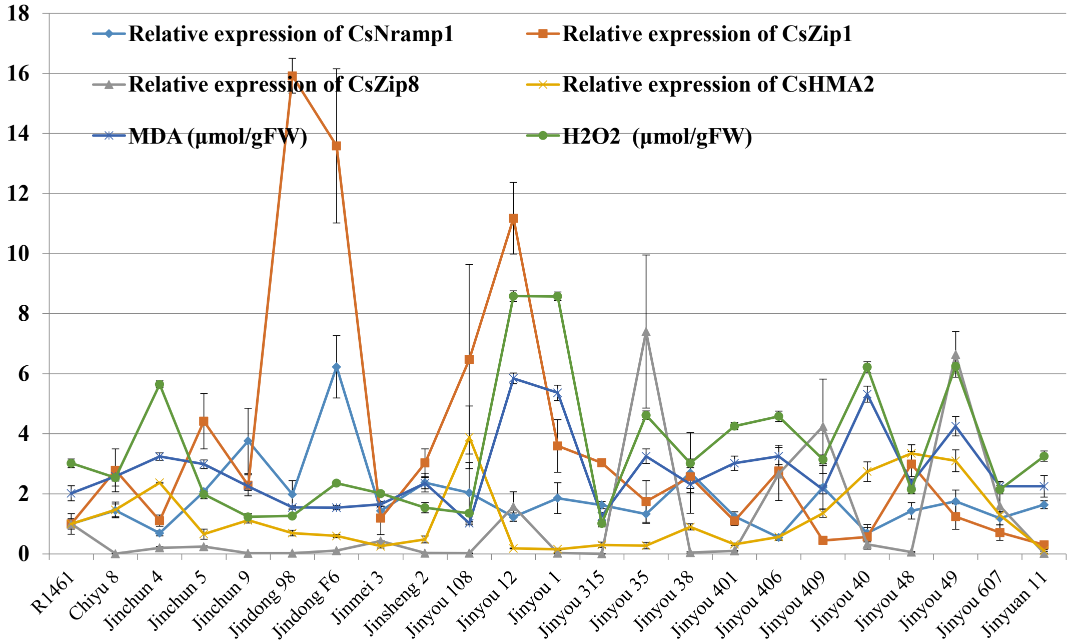

Figure S9. Association analysis of cucumber Cd-related transporter genes expression profiles and Cd induced REDOX reaction. Correlation analysis the expression of *CsNramp1*, *CsZIP1*, *CsZIP8* and *CsHMA2* in 23 cultivated varieties along with MDA and H_2O_2 content in cucumber seedlings. Ten day-old cucumber plants were grown in the vermiculite surround by Yamazaki nutrient solution supplemented with 0 and 0.5 μM Cd for 48h and 4day.

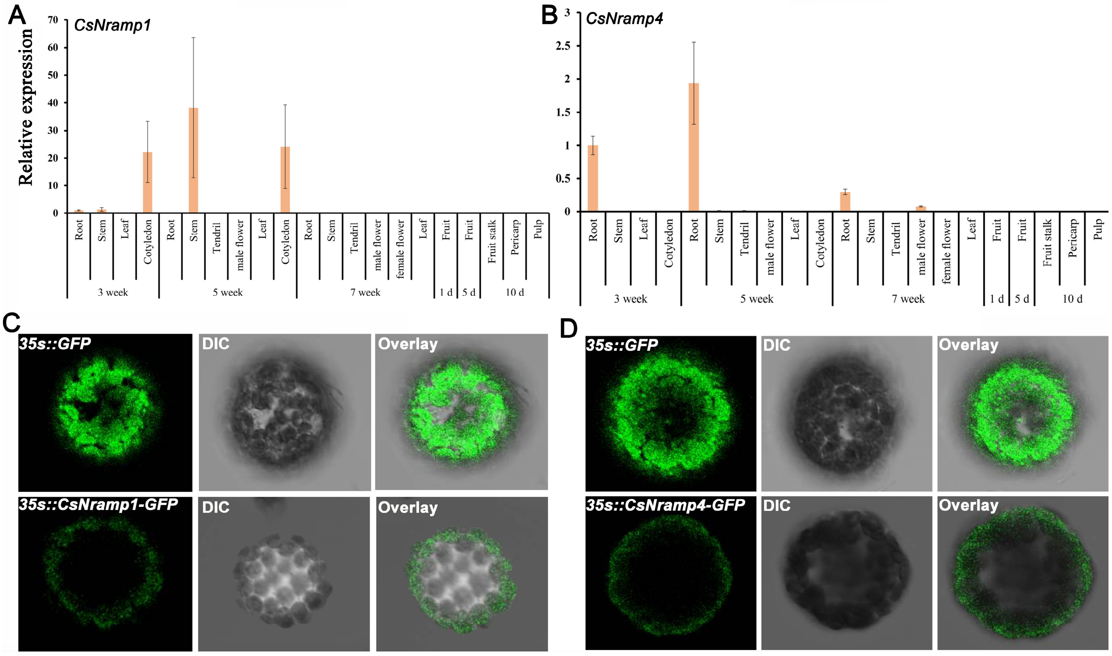

Figure S10. Expression patterns of CsNramp1 and CsNramp4 in wild-type (WT, R1461) under normal conditions. (**A**,**B**) Relative expression in various tissues at different growth stages. Cucumber was grown in a flowerpots until ripening and tissues were sampled. The expression level was determined by quantitative RT-PCR. Vertical bars represent standard deviation of biological triplicate. (**C**,**D**) Subcellular localization of CsNramp1 and CsNramp4 protein in *Arabidopsis thaliana* plants by confocal images. The CsNramp1/4-GFP fusion was constructed and transiently expressed in *Arabidopsis* protoplasts. C: CsNramp1-GFP. D: CsNramp4-GFP.

Table S1. Output data of RNA-seq from four cucumber libraries exposed –Cd and +Cd.

Sample	Raw Reads	Raw Bases	Clean Reads	Clean Bases	Valid Bases	Q30	GC
Normal_1	47475568	7121335200	42352064	6271375740	88.06%	94.05%	45.15%
Normal_2	47180356	7077053400	42083654	6233081174	88.07%	93.99%	45.34%
Normal_3	48862918	7329437700	43061316	6379917756	87.05%	94.07%	45.26%
+Cd1	47352174	7102826100	40923774	6065802690	85.40%	94.19%	44.83%
+Cd2	48036024	7205403600	41682066	6175920371	85.71%	94.35%	44.85%
+Cd3	48870876	7330631400	42371430	6279052572	85.66%	94.36%	44.80%
